# Equilibrium and Nonequilibrium Ensemble Methods for Accurate, Precise and Reproducible Absolute Binding Free Energy Calculations

**DOI:** 10.1021/acs.jctc.4c01389

**Published:** 2024-12-16

**Authors:** Agastya P. Bhati, Shunzhou Wan, Peter V. Coveney

**Affiliations:** † Centre for Computational Science, Department of Chemistry, University College London, London WC1H 0AJ, United Kingdom; ‡ Computational Science Laboratory, Institute for Informatics, Faculty of Science, University of Amsterdam, Amsterdam 1012, The Netherlands; § Advanced Research Computing Centre, University College London, London WC1H 9BT, United Kingdom

## Abstract

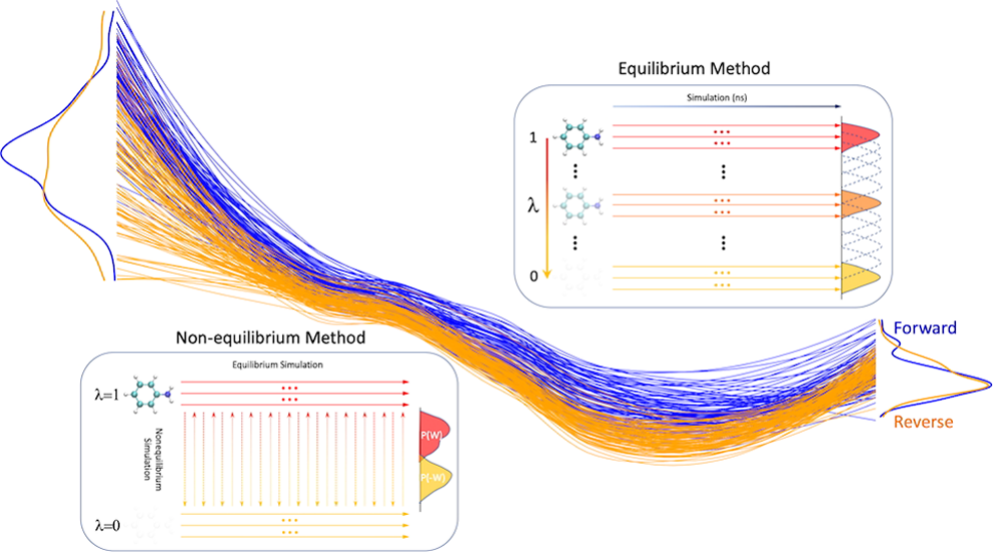

Free energy calculations for protein–ligand complexes have become widespread in recent years owing to several conceptual, methodological and technological advances. Central among these is the use of ensemble methods which permits accurate, precise and reproducible predictions and is necessary for uncertainty quantification. Absolute binding free energies (ABFEs) are challenging to predict using alchemical methods and their routine application in drug discovery has remained out of reach until now. Here, we apply ensemble alchemical ABFE methods to a large data set comprising 219 ligand-protein complexes and obtain statistically robust results with high accuracy (<1 kcal/mol). We compare equilibrium and nonequilibrium methods for ABFE predictions at large scale and provide a systematic critical assessment of each method. The equilibrium method is more accurate, precise, faster, computationally more cost-effective and requires a much simpler protocol, making it preferable for large scale and blind applications. We find that the calculated free energy distributions are non-normal and discuss the consequences. We recommend a definitive protocol to perform ABFE calculations optimally. Using this protocol, it is possible to perform thousands of ABFE calculations within a few hours on modern exascale machines.

## 1 Introduction

In the past decade, structure-based methods have become widespread in drug discovery thanks to a powerful combination of improvements in force field accuracy,^1^ methods, software and hardware, including GPUs.^2^ Due to the extremely limited value of virtual screening methods, binding affinity calculation methods offer a promising alternative. If implemented correctly, they are far more accurate than simple docking scores as they also capture the dynamics of protein–ligand binding including conformational changes and solvation effects. But they are far more computationally expensive and require more wall clock time to compute. The accurate and reliable prediction of protein–ligand binding affinities can evidently play an important role in the drug discovery process.

Relative binding free energy (RBFE) calculations have started to be used seriously in pharmaceutical companies in the past few years.^3,4^ A substantial limitation of RBFE is that it requires the compounds to be structurally and chemically similar to each other, confining its application to lead optimization. To overcome this limitation, other approaches^5^ have been proposed for hit-to-lead discovery, including absolute binding free energy (ABFE) methods. These include approximate methods such as MMPBSA^6,7^ which are faster and less expensive. We have developed an approximate absolute binding affinity prediction method called “ESMACS”^8,9^ that ensures reliable and reproducible free energy predictions and is in use by some pharmaceutical companies.^10−12^ Given the approximations involved, it provides good and reliable ranking of ligand potency which is the most important thing desired during the initial stages of drug discovery. As leads start to emerge, more accurate ABFE predictions become necessary. In that context, the alchemical ABFE approach^13^ is gaining traction these days as it is a theoretically exact method. But it has been regarded as computationally far too expensive, very slow, not easy to implement and automate, and leads to unreliable results when incorrectly implemented.

In the alchemical ABFE approach, the two physical states–one with a ligand bound in the binding site of the protein (bound) and one without (unbound)—are linked via some intermediate states along an alchemical path. Due to the very substantial differences in the two end points (corresponding to *apo* and *holo* proteins), alchemical ABFE is far more demanding to implement reliably compared with alchemical RBFE.^14−16^ To have accurate and precise predictions, many issues need to be taken into account, including uncertainty quantification, reliable sampling of relevant physical and/or nonphysical conformations, identification of “correct” ligand poses, accounting for the conformational changes between *apo* and *holo* protein states and so on.

Because of the computationally demanding requirements of alchemical ABFE calculations, large-scale studies have seldom been performed using such approaches. Hitherto, there have been mainly small-scale studies (involving only a handful of protein–ligand systems) focusing on the accuracy of the approach, including early studies of inhibitors to T4 lysozyme, a model system with specific binding properties comprising small aromatic inhibitors with limited rotational degrees of freedom binding to a small, buried hydrophobic or polar binding site.^15,17,18^ In the past few years, alchemical ABFE has been applied to more complicated molecular systems including bromodomains (BRDs)^19−21^ of the bromodomain-containing proteins, FK506 binding protein 12 (FKBP12)^22,23^ and others.^24−28^

It is only recently that a few studies of absolute free energy calculations using relatively larger data sets (over 100 protein–ligand complexes) have been published.^29−32^ All but one of these studies used the same benchmark data set of protein–ligand complexes and obtained comparable levels of accuracy. Lin et al. performed ABFE calculations for 105 ligand-protein complexes using AMBER20 and demonstrated their capability to run such calculations with cloud computing resources.^30^ Chen et al. presented their ABFE predictions for 199 ligand-protein complexes using Schrödinger’s FEP+ package.^32^ It should be noted that the reliability of FEP+ is questionable as its proprietary nature prevents reproducibility studies to be performed. Further, it employs the REST2 approach that is known to degrade the quality of predicted free energies.^33,34^ Unlike these two studies that employed equilibrium (EQ) simulations to obtain ABFEs, Khalak et al. used nonequilibrium (NEQ) simulations to obtain ABFE predictions for a set of 128 ligand-protein complexes.^31^

None of these studies provide a systematic assessment of the effect of various parameters involved in the complex setup of alchemical ABFE calculations, selecting arbitrary values for such parameters in their calculations without justification. Some authors recognize the importance of performing ensemble simulations to capture the true uncertainty in ABFE predictions by acknowledging that errors obtained through bootstrapping underestimate it (even after using decorrelated samples).^15,21,31,32^ However, even in these handful of studies, the choices of ensemble size (often variously referred to as “independent repeats” or “replicates”) are not systematic but *ad hoc*. Baumann et al.^15^ and Chen et al.^32^ use an ensemble size of 3, whereas Khalak et al.^31^ use 5. Gapsys et al.^21^ vary ensemble size from 3 to 5 for their equilibrium ABFE calculations but use larger ensembles (sizes between 5 and 20) for their nonequilibrium ABFE calculations depending on the system and the free energy component being calculated. However, none of the above-mentioned studies perform any systematic investigation to arrive at an “appropriate” ensemble size, nor do they provide any justification for their choices.

With such varied, arbitrary and unexplainable choices for ensemble size, it is impossible to automate ABFE calculations, which is necessary for large scale applications. None of these studies pay any attention to the distributions of predicted ABFEs which can only be accessed by employing ensemble simulations of sizes larger than these, further rendering the reliability of their predictions questionable. Moreover, none of these large scale studies provides a direct comparison between the ABFE predictions obtained from equilibrium and nonequilibrium simulations. EQ and NEQ methods have only been compared for T4 lysozyme, HSP90 and bromodomain systems previously.^15,21^

In this study, we have used a data set of 219 complexes comprising 31 different proteins and 186 compounds with a variety of scaffolds—the largest data set used so far for validating an alchemical ABFE methodology. Given the substantial differences between the two end-states in an absolute alchemical binding free energy calculation, it is important to control the uncertainties associated with such calculations to ensure reliable and reproducible results.^33,35−38^ We have previously demonstrated that the RBFE results obtained using different molecular dynamics engines (NAMD vs OpenMM) and different methods (TI vs FEP) agree with each other when using ensemble simulations but not when using one-off simulations.^39^ Molecular dynamics (MD) is chaotic; hence performing ensemble simulations is a necessity not an option if robust and reliable predictions are sought.^1,38,40−43^ This is true irrespective of the temporal duration of the MD simulation; see for example our recent study using simulations of duration up to 10 μs where divergence becomes very substantial indeed.^44^ There are several published studies demonstrating that, given a fixed amount of simulation time, ensembles of shorter simulations perform a better sampling than one-off (or a handful of) relatively longer simulations.^8,9,40,45−47^ The theoretical reason is the mixing nature of the dynamics, owing to which each replica rapidly decorrelates from all others, while a single trajectory behaves in the opposite manner, each time step being closely correlated with the preceding one.^40^ Therefore, we apply our TIES approach^33,35,37^ based on ensemble simulations in this study to capture the distributions of predicted binding affinities and perform rigorous uncertainty quantification to ensure reliable results. We obtain free energies by performing ensemble averaging over replicas as stipulated by the principles of statistical mechanics.

Of the two methods being currently advocated, the EQ approach involves performing simulations at fixed intermediate alchemical states such that the system remains in equilibrium throughout, whereas the NEQ approach relies on rapidly varying the alchemical state of the system such that the system is driven out of equilibrium during the simulation. We have employed both approaches in this study and we directly compare them against each other over the entire data set. We critically assess the various features of each approach and their relative advantages including recent claims that the NEQ approach is computationally more efficient.^21,31^ In this respect, the present study is analogous to a similar one we recently reported for the RBFE methodology.^16^

We provide a systematic account of the effect of various parameters involved in these calculations and recommend optimal values for each. The size of the ensembles, the duration of the simulations and the number of intermediate states are among the numerous parameters that need to be chosen appropriately. In addition, specific to the NEQ approach, there are numerous additional parameters such as the number and length of the fast alchemical transitions that need to be selected. Furthermore, the overlap in phase space and the dissipation work values arising during the fast alchemical transitions must also be carefully controlled.^15,16^ None of these aspects has been treated systematically hitherto.

Equipped with the findings from this work, we are able to provide definitive recommendations on the optimal implementation of the alchemical ABFE method in order to ensure reproducible, accurate and precise results at lowest cost. Finally, our ensemble approach improves sampling while allowing one to run all the required ABFE simulations concurrently, thus minimizing the wall-clock time requirements, such that the entire ABFE protocol can be run in less than 2 h. This is relevant for large scale applications as thousands of ABFE predictions can be made within a few hours given sufficient resources and/or access to modern supercomputers.

## 2 Methods

In this section, we describe the alchemical approach employed for ABFE calculations in this study for both EQ and NEQ methods. We also describe our data set and the details of the protein–ligand model preparation and simulations performed. Our approach to uncertainty quantification based on ensemble simulations is explained. Finally, we also provide an estimate of the computational costs associated with various recommended ABFE protocols and the corresponding time-to-solution.

### 2.1 Double Decoupling Method

**Figure 1:**
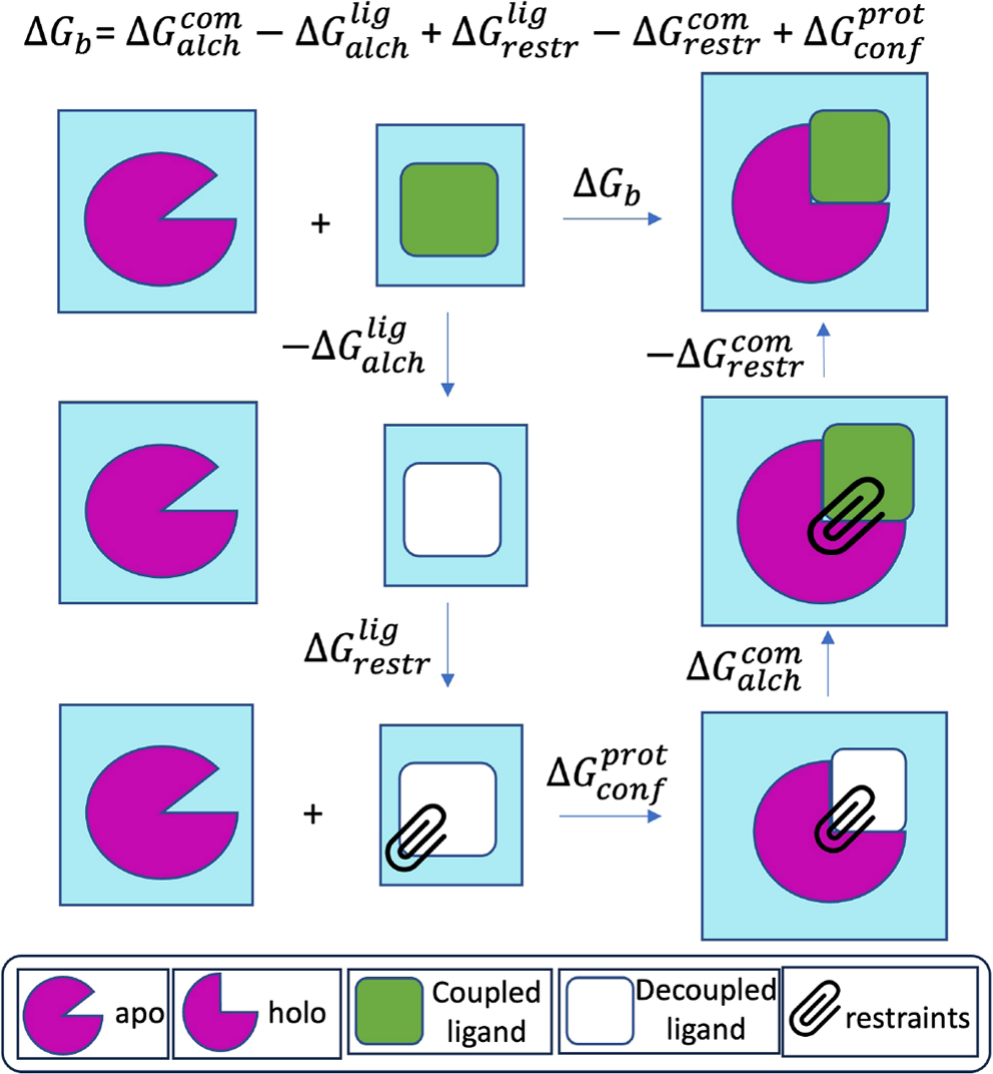
Thermodynamic cycle employed for the alchemical absolute binding free energy calculations. The process of binding is divided into a series of nonphysical transformations each with an associated Δ*G*: ligand decoupling from solvent (−Δ*G*_alch_^lig^), restraining the decoupled ligand within the binding pocket of protein (Δ*G*_restr_^lig^ + Δ*G*_conf_^prot^) and ligand coupling in the protein environment (Δ*G*_alch_^com^) while relaxing the restraints (−Δ*G*_alch_^com^). The binding free energy (Δ*G*_b_) is given by the sum of all these terms. Ensemble sizes need to be varied for various steps in this cycle to keep errors under control.

We employ the double decoupling scheme^48,49^ using the thermodynamic cycle shown in Figure 1. The physical process of binding is decomposed into several alchemical steps with a free energy value (Δ*G*) associated with each step. The binding affinity of the ligand-protein complex (Δ*G*_b_) is given by the sum of these individual Δ*G* values. The process begins with the decoupling of the ligand in solvent such that the electrostatic and van der Waals interactions between ligand and solvent are turned off (−Δ*G*_alch_^lig^). This is followed by restraining the decoupled ligand within the binding site of the protein using a set of restraints proposed by Boresch et al.^50^ (Δ*G*_restr_^lig^ + Δ*G*_conf_^prot^). It should be noted here that we add an additional free energy term in this step (Δ*G*_conf_^prot^) that captures the change in protein conformation from *apo* state to *holo* state. This term is usually ignored and leads to systematic errors in the predicted ABFEs. Finally, the ligand is coupled with the protein environment by turning on ligand-protein electrostatic and van der Waals interactions while relaxing the restraints (Δ*G*_alch_^com^ – Δ*G*_restr_^com^). The final two steps of coupling the ligand and removing its restraints may be performed either separately (coupling followed by relaxation) or simultaneously. We show that both yield almost identical results while the latter reduces the computational costs substantially (see Figure S3 and the related section in the Supporting Information for details). The terms Δ*G*_alch_^com^, −Δ*G*_alch_^lig^ and −Δ*G*_restr_^com^ are obtained using simulations, whereas Δ*G*_restr_^lig^ is calculated analytically as proposed by Boresch et al.^50^ However, it is not straightforward to obtain Δ*G*_conf_^prot^; we propose a way to estimate it as discussed in the Results section. We compare two distinct approaches—equilibrium (EQ) and nonequilibrium (NEQ)—as described in Sections 2.2 and 2.3. The above thermodynamic cycle is used with both these approaches and the binding affinity is given by the following equation:
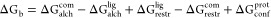
1

### 2.2 Equilibrium Approach

This approach involves dividing an alchemical transformation into several steps with fixed values of the alchemical parameter, λ (referred to as λ-windows hereafter), and performing simulations at each λ-value such that the system always remains in equilibrium. We use thirteen λ-windows for ligand (de)coupling both in water and protein environment with λ = {0.0, 0.01, 0.05, 0.1, 0.2,..., 0.9, 1.0}. Herein, the λ scheme used is such that the (de)coupling of the van der Waals and the electrostatic interactions are differentiated, the latter being perturbed faster than the former as explained below. The van der Waals interactions are perturbed linearly across the full range of λ values such that λ = 0/1 corresponds to the van der Waals interactions fully turned off/on, respectively. On the other hand, the electrostatic interactions remain extinguished between λ = 0 and 0.45 and are perturbed linearly from λ = 0.45 to 1. Further, we use 12 λ-windows for the removal of restraints on the ligand in the protein environment (when performed separately from ligand coupling) with λ = {1.0, 0.75, 0.5, 0.3, 0.2, 0.15, 0.1, 0.075, 0.05, 0.025, 0.01, 0.0}. Both these series of λ-windows are more closely spaced near λ = 0 due to the steeper energy gradient in the corresponding region of the phase space. When ligand coupling and removal of restraints in the protein environment are performed simultaneously, we use 15 λ-windows = {0.0, 0.01, 0.05, 0.1, 0.2,..., 0.9, 0.95, 0.99, 1.0} (with the strength of restraints varying as 1−λ). Throughout the simulations at each λ-window, the energy gradient with respect to λ (∂*H*/∂λ, where *H* is the Hamiltonian) is collected which is then postprocessed to obtain the final Δ*G* using the following equation:
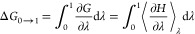
2It should be noted here that the angular brackets in the above equation represent ensemble averaging which we perform over replicas. We would also like to remind readers here that different alchemical free energy estimators, such as TI, FEP, and MBAR (a generalized version of BAR), yield comparable results when employing ensemble simulations.^35,39^ Consequently, there is no reason to prefer any one estimator over the other and the claims that BAR (or MBAR) is better than TI is unfounded. We use our in-house TIES software for the analysis of simulation output to obtain free energies.^51,52^

### 2.3 Non-Equilibrium Approach

In this sequential approach, the first step is to create a set of conformations for both physical end-states (corresponding to λ = 0 and 1) by performing equilibrium simulations at each. Thereafter, fast transitions are initiated from each conformation of either end-state, transforming the system into the other end-state through the alchemical space. The transitions initiated from the λ = 0 end-state conformations are called “forward” transitions, whereas those initiated from the λ = 1 end-state conformations are termed “reverse” transitions. Such fast transitions drive the system out of equilibrium; hence it is a nonequilibrium approach. The dissipation work done to perform each transition is obtained by integrating ∂*H*/∂λ over the course of the transition (referred to as “work values” hereafter). Δ*G* values are computed from the work value distributions in both forward and reverse directions using a maximum likelihood estimator^53^ based on the Crooks Fluctuation Theorem^54^ using an adapted version of pmx.^55^ It should be noted that the NEQ approach by design leads to the occasional occurrence of extreme work values in the forward direction when the ligand is coupled in the protein environment. Such extreme values are inevitable in NEQ simulations and compromise accuracy and precision of this approach, which undermines its reliability. We discuss this aspect in detail in the Supporting Information (see Table S1, Figures S1 and S2, and the related section). The results presented in this study have been obtained after ignoring all work values >1000 kcal/mol.

### 2.4 Protein–Ligand Data Set and Structure Preparation

The data set used in this study is a collection of several protein–ligand systems, some of which have been previously studied by various groups while the others are new. This includes a set of 7 proteins and 128 ligands that were part of our previous large scale study of RBFE calculations:^33^ CDK2, cMET, Galectin, JNK1, P38, PDE2, and TYK2. This same set has also been studied using the NEQ approach for ABFEs^31^ and its subset using Schrödinger’s FEP+.^32^ Further, we include a set of bromodomains studied previously using the EQ approach for ABFEs: (a) bromodomain 1 of BRD4 binding to a set of 8 ligands^19^ (referred to as “BRD4–1” in this study), (b) 22 bromodomains binding to a set of 3 different compounds (bromosporine, RVX–OH and RVX-208) amounting to a total of 34 ligand-protein complexes.^20^ A subset of the above bromodomain systems were also studied using the NEQ approach for ABFEs.^21^ Further, we included three systems that we studied previously using ESMACS for ABFEs and TIES for RBFEs but have not been studied for ABFEs using the alchemical approaches so far: bromodomain 1 of BRD4 binding to 9 different ligands^10^ (referred to as “BRD4–2” in this study), TrkA binding to 16 ligands^11^ and ROS1 binding to 24 ligands.^56^ Overall, our data set comprises 219 ligand-protein complexes spanning 31 different proteins (including 22 bromodomains) and 186 ligands. It is the largest studied so far using alchemical ABFE methodologies.

The input structures were obtained from our previous large scale RBFE study^33^ along with the Protein Data Bank (PDB)^57^ corresponding to PDB IDs drawing on previous studies referred to above. The *apo* structures were obtained from PDB using the following PDB IDs: 2OSS(58) for BRD4–2, 1H27(59) for CDK2, 1R1W(60) for cMET, 3ZSL(61) for Galectin, 3O17 for JNK1, 1WFC(62) for P38, 4HTZ(63) for PDE2 and 4F0I(64) for TrkA. For structures downloaded from PDB, all crystal water molecules within 5 Å of the protein were retained. Protonation states and tautomeric forms of histidine residues were kept consistent with previous studies. Any missing residues are modeled using ModLoop.^65^ GAFF (v2)^66^ parameters were used to prepare ligand molecules with charges calculated using AM1-BCC model.^67^ The AMBER ff14SBonlysc or ff14SB^68^ force field was used to parametrize all proteins. Our systems were solvated in an orthorhombic TIP3P^69^ water box with at least a 14 Å solvent in all directions. Sodium and chloride ions were used to neutralize the system electrostatically employing Joung-Cheatham ion parameters.^70^ AmberTools20^71^ was used to perform parametrizations and prepare all models.

### 2.5 Simulation Details

All simulations were performed using NAMD.^72^ For all equilibrium simulations (that is, those with fixed λ values in both the EQ and the NEQ approach), the ligand in the protein environment was first energy minimized, followed by a 20 ps NVT equilibration and a 2 ns NPT equilibration. Pressure and temperature were maintained at 1 atm and 300 K using a Berendsen barostat (compressibility of 4.57 × 10^–5^ bar^–1^ and relaxation time of 100 fs) and a Langevin thermostat (damping coefficient of 5 ps^–1^), respectively. The production run time was taken to be 10 ns after a systematic and extensive analysis (as detailed in the Results section). A time step of 2 fs was used. When a ligand in the water environment was simulated, the entire protocol remained the same except that the NPT equilibration step was only 1 ns long. During both minimization and equilibration steps, protein backbone atoms were initially constrained to their initial positions and were slowly allowed to relax. Periodic boundary conditions were employed with long-range electrostatics handled by the Particle Mesh Ewald (PME) method.^73,74^ A nonbonded cutoff of 12 Å was used. The van der Waals interactions were smoothly switched off between 10 and 12 Å, being linearly perturbed between λ value 0 and 1. The standard NAMD soft-core potential^75,76^ was used for the van der Waals terms with the radius-shifting coefficient of 5 Å^2^ to avoid singularities. While the coordinates were recorded every 10 ps, energy derivatives with respect to λ were recorded every 2 ps.

For nonequilibrium transitions, simulation settings remain the same; the simulations are initiated from equidistant conformations chosen from production runs of the equilibrium simulations at the two end-states. The number of conformations chosen in each direction (in other words, the number of forward and reverse transitions) and their lengths are NEQ specific-parameters that cannot be chosen arbitrarily as done in all previous ABFE studies using the NEQ approach. The only exception is the study by Gapsys et al.^21^ where the transition length was varied up to 20 ns to show that 500 ps was optimal for a small set of 7 ligands. We have performed a systematic investigation of the effect of these parameters on ABFE calculations using a large data set and recommended optimal values for each as discussed in the Results section. For this study, we have generally used our recommended “standard” protocol (see the Discussion section for detail). This requires performing 500/250 nonequilibrium transitions in each direction with protein/water environment starting from equidistant equilibrium conformations (50 per replica chosen at an interval of 200 ps taking an ensemble size of 10/5) per ABFE prediction. Furthermore, it requires differential transition lengths in the two directions for simulations with protein: 800 ps for forward and 400 ps for reverse transitions.

### 2.6 Uncertainty Quantification

Given the chaotic nature of MD,^40−42^ it is necessary to perform ensemble simulations in order to control uncertainties associated with its predictions and ensuring reproducibility of results, thereby making them reliable.^1,35,38,,44^ Therefore, in this study we employ our TIES approach^33,35,37^ which involves performing ensemble simulations. The size of the ensemble is a parameter whose value cannot be arbitrarily chosen as is the usual practice among those research groups that deign to perform multiple independent simulations (3 is the most common choice).^31,32^ In fact, the ABFE thermodynamic cycle in Figure 1 is a workflow so each step needs separate considerations as far as ensemble size is concerned in order to keep the overall uncertainty under control. This means that, for the ligand decoupling step in solvent alone, relatively smaller ensembles could be used without compromising the accuracy and precision of predictions. Gapsys et al.^21^ varied ensemble size from 3 to 5 for the EQ method and 5 to 20 for the NEQ method (in fact, 20 was only used to calculate Δ*G*_alch_^lig^ for the bromosporine system) but their choices were arbitrary, system-dependent and without any justification or systematic investigation. Here, we perform a systematic investigation of the effect of ensemble size on alchemical ABFE predictions by varying it from 1 to 100 and determine an appropriate value for optimal performance, that enables achieving chemical accuracy (<1 kcal/mol) and desired precision (≤0.5 kcal/mol). Further, we point out that it should be considered a flexible parameter with adjustments made whenever necessary. Based on this analysis, we determine that an ensemble size of 10 is the most appropriate for alchemical ABFE simulations in the protein environment and use this value as a standard in all our calculations. However, for the ligand decoupling in water, an ensemble size of 5 is found sufficient due to lesser fluctuations in energy derivatives across replicas as also shown in our previous study.^35^ It is noteworthy that larger ensembles are required for a thorough investigation of non-normal distributions.

For the EQ approach, we employ our TIES protocol (successfully used for RBFEs^33,35,37^) that involves performing ensemble simulation at each intermediate alchemical state followed by ensemble averaging of the bootstrapped energy derivatives so obtained. Such averaged energy derivatives are then integrated using the principles of stochastic calculus to get the final Δ*G* along with associated uncertainties using eq 2. In the case of the NEQ approach, we adapt TIES to obtain the uncertainties associated with predicted free energies. We use the maximum likelihood estimator^53^ to obtain a Δ*G* value using the forward and reverse work values corresponding to all equilibrium replicas collectively at each end-state. However, the associated uncertainty reported is the standard error of Δ*G* values obtained using work values corresponding to individual equilibrium replicas at each end-state. Thus, we generate an ensemble of Δ*G* values with size equal to the size of the ensemble simulation performed at each end-state in order to estimate uncertainty.

### 2.7 Computational Costs and Time to Solution

Alchemical ABFE calculations are computationally intensive. Table 1 summarizes the computational costs associated with them using both the “standard” as well as the “minimum” protocols recommended in this study (see Table 8 in Discussion). Using the standard recommended protocol (employed in this study), the simulation time requirement for a single ABFE calculation using the NEQ approach is only about 10% cheaper than the EQ approach. The aggregate simulation time invested for this entire study covering both EQ and NEQ methods and including all simulations required for the various systematic analyses is over 3 ms.

**Table 1:** Overview of Computational Requirements for EQ and NEQ Methods of ABFE Calculations^a^

	standard		minimum	
component	EQ	NEQ	EQ	NEQ
Δ*G*_alch_^com^ (ns)	1500	800	375	200
Δ*G*_restr_^com^ (ns)		800		200
Δ*G*_alch_^lig^ (ns)	650	300	325	150
overall (μs)	2.15	1.9	0.7	0.55

a The number of λ-intermediates in case of EQ calculations is taken to be 15 for the complex leg and 13 for the ligand leg. In the case of NEQ, the computational requirements for Δ*G*_restr_^com^ are taken equal to those for Δ*G*_alch_^com^ for comparison. Although in this study, Δ*G*_restr_^com^ from the EQ protocol (calculated using 12 λ-intermediates) has been reused in the case of NEQ.

One of the advantages of the ensemble approach is that it substantially improves sampling in order to control uncertainties efficiently without adding to the wall clock time to solution. The entire ABFE protocol (minimum, standard or further extended) can be performed in the same amount of wall clock time as required for a single simulation by executing all replicas concurrently. With modern supercomputing hardware, it is not uncommon to achieve a performance of around 200 ns/day using a single GPU for typical protein–ligand systems. Therefore, it is possible to run the full protocol in less than 2 h. The only requirement is the availability of sufficient resources. In fact, we performed around 150 ABFE EQ calculations using our standard protocol within a few hours on Summit (Oak Ridge National Laboratory) in November 2021 when we had access to the full machine for 36 h. Given that our ensemble approach is embarrassingly parallelizable on larger machines, we are able to perform thousands of such calculations on the world’s first exascale machine, Frontier, within the same time frame.

## 3 Results

We performed ABFE calculations on 219 ligand-protein complexes comprising 186 ligands and 31 different protein targets (including 22 bromodomains). This is the first ever application of both equilibrium (EQ) and nonequilibrium (NEQ) alchemical approaches to predict absolute binding free energies (ABFEs) for an identical set of ligand-protein complexes at such a large scale, thereby permitting their direct comparison. We would like to point out here that all the results obtained from the NEQ approach discussed in this section use the “standard” recommended protocol (as detailed in the Discussion section). It requires differential NEQ transition lengths in the forward (800 ps) and the reverse (400 ps) directions; hence it will be referred to as the “600 ps” protocol hereafter. Further, we have also performed NEQ calculations using four different transition lengths (400 ps, 800 ps, 1 and 2 ns; same length used in both directions) for the entire data set whose predictions have been included in the Supporting Information and are referred to at appropriate places in the main text. As already mentioned in Section 2.3, in case of the NEQ approach, we report the occurrence of extreme work values (>1000 kcal/mol) for forward transitions when the ligand is coupled in the protein environment. The origin and extent of such extreme work values is discussed in detail in the Supporting Information (see Table S1, Figures S1 and S2, and the related section). Such work values degrade the accuracy and precision of ABFE predictions. It is worth noting, however, that the accuracy is not affected by such extreme values when ensemble simulations are employed, although uncertainties increase substantially. Therefore, all results reported in this study have been obtained after ignoring such extreme work values.

**Figure 2:**
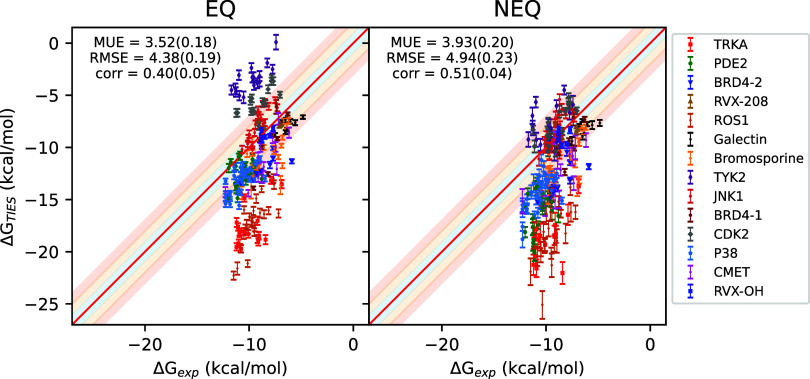
Correlation plots for absolute binding free energies from the equilibrium (EQ) as well as nonequilibrium (NEQ; 600 ps) alchemical methods against experimental data. The solid red line denotes the perfect correlation, whereas the blue, orange and red shaded regions represent ±1, ±1–2, and ±2–3 kcal/mol ranges.

Figure 2 displays the correlation between raw Δ*G* predictions from EQ as well as NEQ approaches against experimental values using the 600 ps protocol. Statistical measures of accuracy are displayed in the inset textbox. Figure S4 contains similar plots for other transition lengths. Before discussing the results further, we would like to point out here that, remarkable as it may seem, experimental values of BFEs are generally reported as single values without any error bars. For instance, Kramer et al.^77^ found that, out of the total 261k protein–ligand systems with *K*_*i*_ measurements included in the ChEMBL data set (version 12), only about 10% had more than one reported *K*_*i*_ measurements available. Of these, more than half were either duplicate entries or cases where multiple measurements came from different publications. This means that less than 5% of the total systems had multiple *K*_*i*_ measurements reported in the same publication. Even for this small subset, multiple measurements mostly correspond to reports on assay optimizations or different stereoisomers. Given the lack of ensembles, the reliability of experimental binding affinity estimates is also questionable. Ross et al.^78^ recently assessed the extent of reproducibility in experimental Δ*G* estimates and reported that RMSE between ABFE measurements from independent experiments is on average 0.9 kcal/mol (using different assays) which could be as high as 2 kcal/mol in some cases. Therefore, this is the limit of the accuracy achievable by any computational method predicting absolute ABFEs. We recently reported non-normality in experimental BFE distributions^79^ which obviously cannot be accounted for using one-off predictions. Further, the lack of uncertainty in experimental BFEs is responsible for bias in the least-squares regression metrics undermining their reliability.^33,80^

Mean unsigned error (MUE), root mean squared error (RMSE) and Pearson’s *r* for the overall data set are 3.52(0.18) kcal/mol, 4.38(0.19) kcal/mol, and 0.40(0.05) respectively using the EQ approach, whereas 3.93(0.20) kcal/mol, 4.94(0.23) kcal/mol, and 0.51(0.04) respectively using the NEQ approach. At face value, these figures do not appear encouraging; however, looking carefully at the correlation plot, systematic errors in predicted Δ*G* values are clearly visible that heavily compromise the overall accuracy of predictions. This is a known issue in ABFE calculations as the two end-points of the ligand coupling alchemical transformation use the same protein conformation, while the two end-points represent *apo* and *holo* conformations of the protein which may be substantially different (including differences in their protonation and/or tautomeric states).^21,31,32^ Indeed, these systematic errors may also partly (or even fully) correspond to multiple modes of ligand binding within the protein environment or to other factors such as differences in water occupancy, and may appear exaggerated due to lack of reported non-normal experimental BFE distributions (see the Supporting Information for more details). Such a difference in protein conformation and/or ligand binding mode is not directly accounted for in these ABFE calculations, leading to systematic errors in the predicted Δ*G*s, an issue present in both EQ and NEQ methods. For the raw predictions, MUE and RMSE values obtained using EQ are better than those obtained using NEQ. The correlation is, however, better using NEQ due to a wider distribution of Δ*G* values in case of EQ. It must be noted that the correlation coefficient is a meaningful metric only when absolute values of Δ*G* are compared which is not true in our case due to the prevalence of systematic errors in predicted values that have widely varying magnitudes across systems. On ignoring the systems with large magnitudes of systematic errors (>2 kcal/mol for TYK2, CDK2, and PDE2), the revised MUE, RMSE and *r* are 3.44(0.21) kcal/mol, 4.41(0.24) kcal/mol and 0.58(0.04) respectively using the EQ approach, whereas 4.15(0.24) kcal/mol, 5.20(0.27) kcal/mol and 0.58(0.04) respectively using the NEQ approach. For this subset of data, EQ still has better MUE and RMSE values than NEQ, but the correlation is also comparable now. Further, the ranges of RMSE values across systems are 0.54–7.90 and 0.95–8.98 kcal/mol respectively for EQ and NEQ approaches. For individual systems, RMSE values using the EQ approach are better for 11 out of 14 systems as compared to the NEQ approach.

In this study, we propose a way to implicitly account for such systematic errors (denoted as Δ*G*_conf_^prot^) in predicted ABFEs. We assume for convenience that such errors for all ligands binding to the same pocket of the target protein are approximately the same. Therefore, we aim to determine a constant that may be used as an empirical estimate of the average error for all such ligands. Such an empirical estimate of Δ*G*_conf_^prot^ can be used to adjust the predicted Δ*G* values. In this case, we use the mean signed error (MSE) value for all ligand-protein complexes belonging to the same system as the empirical estimate of Δ*G*_conf_^prot^. A similar approach has also been independently proposed by Chen et al.^32^ where they used the difference between the average raw predicted Δ*G*s and the average experimental Δ*G*s.

**Figure 3:**
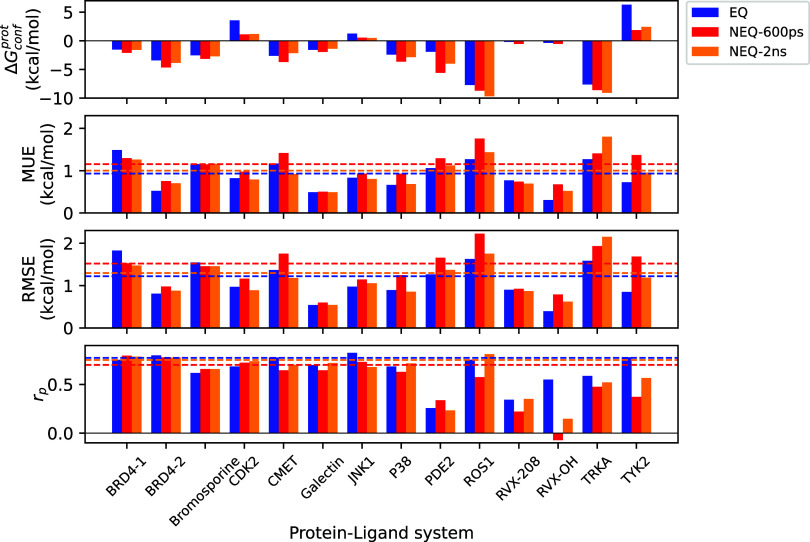
Summary of ABFE predictions using EQ and NEQ methods for each protein–ligand system. NEQ predictions using 600 ps and 2 ns have been included. Δ*G*_conf_^prot^, MUE and RMSE (after adjustment with Δ*G*_conf_^prot^) and Pearson’s *r* (*r*_p_) are displayed and compared using different methods for all systems. The dashed lines (using the same color code as bar plots) denote corresponding values for the full data set. Further details are provided in Table S2. It is clear that the EQ method is more accurate than the NEQ method. All energies are in kcal/mol.

Figure 3 and Table S2 contains the values of such errors for all systems studied using both approaches. It can be seen that the direction of the error (sign of Δ*G*_conf_^prot^) is system-dependent and fully consistent between EQ and NEQ methods. As explained in the Supporting Information, a negative value indicates that the dominant contributor to these systematic errors are the conformational differences between *apo* and *holo* states of the protein. On the other hand, a positive value suggests that the protein structures in *apo* and *holo* states are similar (or identical) but other factors mainly contribute to the systematic errors. Eleven out of 14 systems studied here (exceptions are CDK2, TYK2, and JNK1) have negative Δ*G*_conf_^prot^ values which means that *apo* and *holo* conformational differences are indeed responsible for the said systematic errors in a majority of cases.

Interestingly, the magnitude of errors is consistently lower in the case of the EQ approach when their sign is negative, whereas the opposite is true for positive Δ*G*_conf_^prot^ values. This makes sense given that the system is allowed to relax at each intermediate alchemical state while staying in equilibrium in the case of the EQ approach, which is not achievable during the short out-of-equilibrium simulations in the case of the NEQ approach. In case of negative errors, two conformations of the protein in its *apo* state (true/experimental apo structure versus holo structure with a ghost ligand) are connected by a minimal energy barrier and such a relaxation in the EQ approach allows the sampling of experimental *apo* conformation (the “global minimum”), thereby narrowing the difference between experimental and calculated free energies in the apo state.

However, in the case of positive errors, the energy barrier between the two *holo* conformations (such as those corresponding to two different binding modes and/or water occupancy states) may be high such that a simple relaxation may only be able to partially sample one or a few high energy intermediate states leading to a magnification of errors. This issue related to the discrepancy in *holo* conformations is not specific to the alchemical ABFE methods, but is a general issue that occurs with any MD based method (free energy or otherwise). All such methods are sensitive to the starting structure used and their predictions heavily depend on it. Further, the apparent fact that EQ intensifies the magnitude of (positive) errors does not imply that NEQ is a better approach. It simply means that the NEQ approach is incapable of overcoming the energy barrier to sample other possible conformations, resulting in biased predictions. On the other hand, the EQ approach, by design, enables the crossing of such energy barriers and may reduce or even eliminate the bias in predictions given sufficient sampling time. The uncertainty in Δ*G*_exp_^78^ is also partially responsible for these errors. This means that there might be scope for improvement in these correlations had we access to more comprehensive data on the distribution of Δ*G*_exp_.^79^

**Figure 4:**
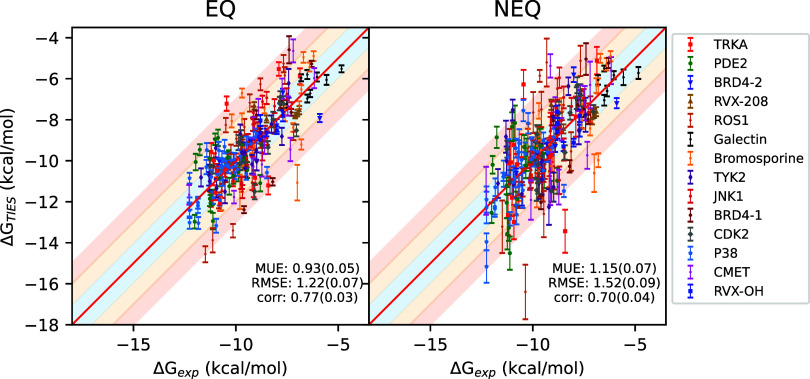
Correlation plots for absolute binding free energies from the equilibrium (EQ) as well as nonequilibrium (NEQ; 600 ps) alchemical methods against experimental data after adjusting for Δ*G*_conf_^prot^. The solid red line denotes the perfect correlation, whereas the blue, orange and red shaded regions represent ±1, ±1–2, and ±2–3 kcal/mol ranges.

Figure 4 displays the correlation plot for all ligand-protein complexes studied using the 600 ps protocol after adjustment using Δ*G*_conf_^prot^ for both EQ and NEQ approaches. Figure S5 captures corresponding plots for other transition lengths. MUE and RMSE values fall to 0.93(0.05) and 1.22(0.07) kcal/mol respectively, and Pearson’s *r* goes up to 0.77(0.03) for the EQ approach. Similarly, the updated MUE, RMSE, and Pearson’s *r* values for the NEQ approach are 1.15(0.07) kcal/mol, 1.52(0.09) kcal/mol and 0.70(0.04) respectively. It should be noted that such an adjustment scheme can only be applied when experimental data is available which is usually not the case in prospective applications such as drug discovery campaigns. However, adjusting Δ*G* values by subtracting a constant from them does not affect the ranking of the ligands in terms of their binding potency. Thus, the application of this adjustment scheme is not in fact necessary when one is primarily concerned with ranking as in drug discovery scenarios.

Figure 3 and Table S2 capture the number of complexes, Δ*G*_conf_^prot^, MUE, RMSE, and Pearson’s *r* for each system studied separately, using both EQ and NEQ approaches. RMSE values (after adjustment) across systems range between 0.39–1.83 and 0.60–2.23 kcal/mol respectively for EQ and NEQ approaches. Pearson’s correlation coefficients fall between 0.26 and 0.82 for EQ and between −0.07 and 0.80 for NEQ across all systems. For the full data set, the EQ approach agrees better with the experimental data as compared to the NEQ approach with differences between their MUEs and RMSEs being 0.22 and 0.30 kcal/mol, respectively. On comparing individual systems, all but two systems have RMSEs differing between EQ and NEQ approaches by less than 0.5 kcal/mol (after adjustment with Δ*G*_conf_^prot^). So, the difference in accuracy between the two methods is primarily due to ROS1 and TYK2, where the NEQ method performs much worse compared to the EQ method (RMSEs differing by 0.60 and 0.84 kcal/mol respectively). The difference in precision of the two approaches is even more stark: the numbers of complexes with uncertainties greater than 0.5 and 1 kcal/mol are 5.48% and 0 for the EQ approach, whereas 51.15 and 6.85% for the NEQ approach. Further, the range of uncertainties is 0.14–0.87 and 0.13–1.33 kcal/mol for EQ and NEQ approaches, respectively.

**Figure 5:**
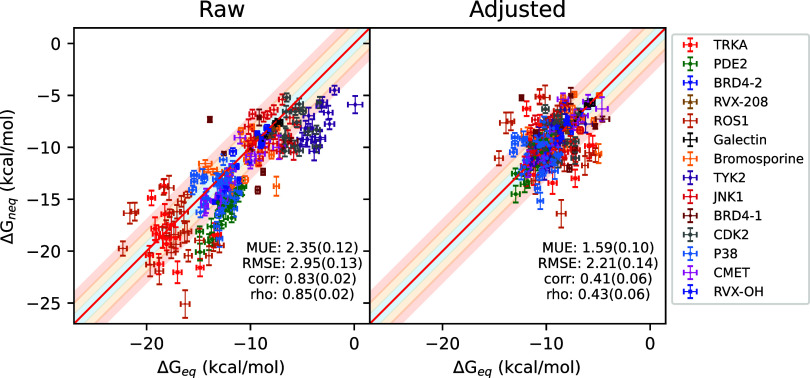
Correlation plots for absolute binding free energies from the nonequilibrium method (600 ps) against those from the equilibrium method for both raw predictions as well as adjusted for Δ*G*_conf_^prot^. The solid red line denotes the perfect correlation, whereas the blue, orange, and red shaded regions represent ±1, ±1–2, and ±2–3 kcal/mol ranges.

Figures 5 (600 ps), S6 and S7 (400 ps to 2 ns) display a direct comparison of results obtained from EQ and NEQ approaches for both raw as well as adjusted data. Given the MUE and RMSE values and looking at the comparison of the raw data, it is clear that the systematic errors in predicted ABFEs have different magnitudes using the two approaches for some systems. Even after adjusting the results using Δ*G*_conf_^prot^ for both approaches, the results do not agree too well with MUE and RMSE of 1.59(0.10) and 2.21(0.14) kcal/mol as well as Pearson’s *r* and Spearman’s ρ of 0.41(0.06) and 0.43(0.06) respectively (see Figure 5). These findings, in conjunction with the fact that EQ results agree better with the experimental values, lead to the conclusion that the EQ approach is preferable over the NEQ approach as it is more accurate and precise.

### 3.1 Non-Gaussian Nature of Predicted Free Energies

**Figure 6:**
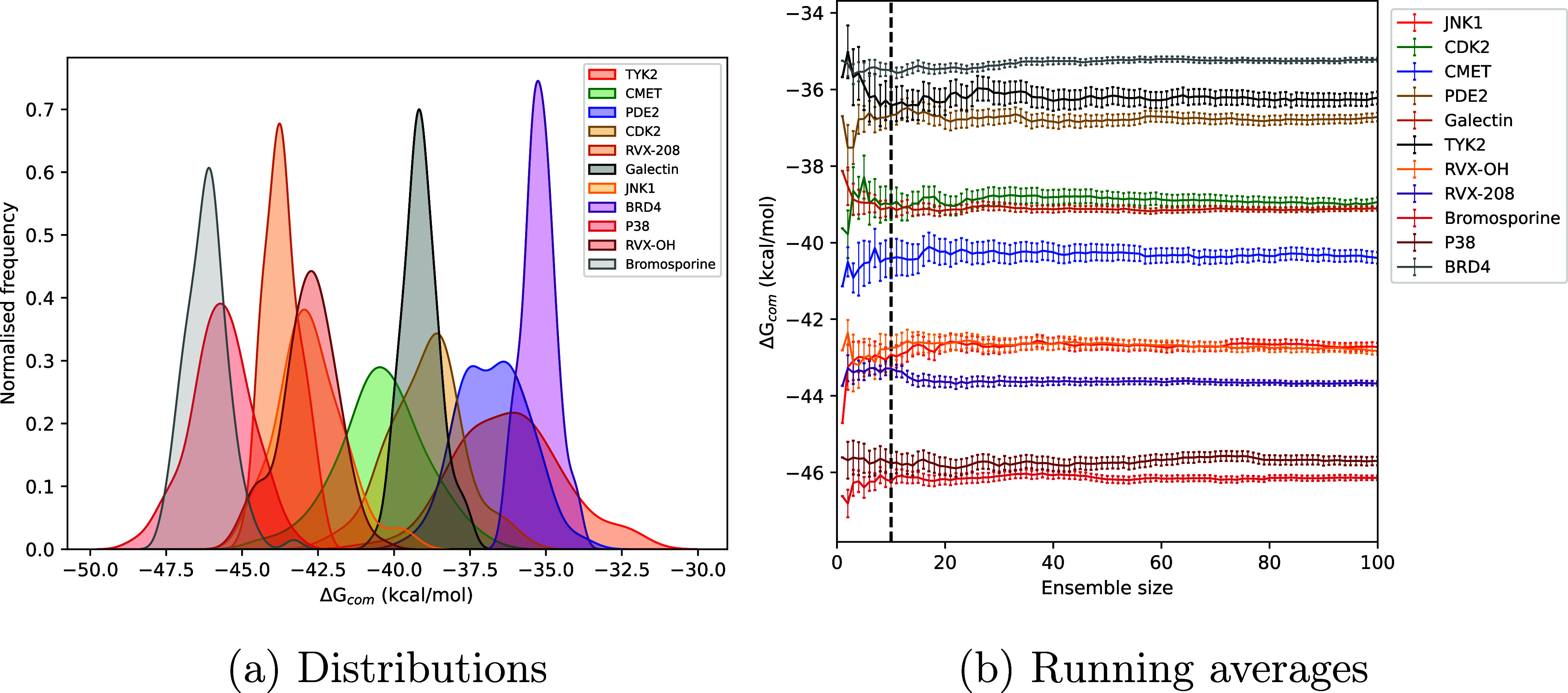
(a) Normalized frequency distributions of predicted alchemical ABFEs for the equilibrium approach using ensemble size 100 for a subset of ligand-protein complexes depicting their non-Gaussian nature. (b) Running averages of predicted ABFEs varying with the ensemble size. The dashed vertical line denotes ensemble size 10.

**Figure 7:**
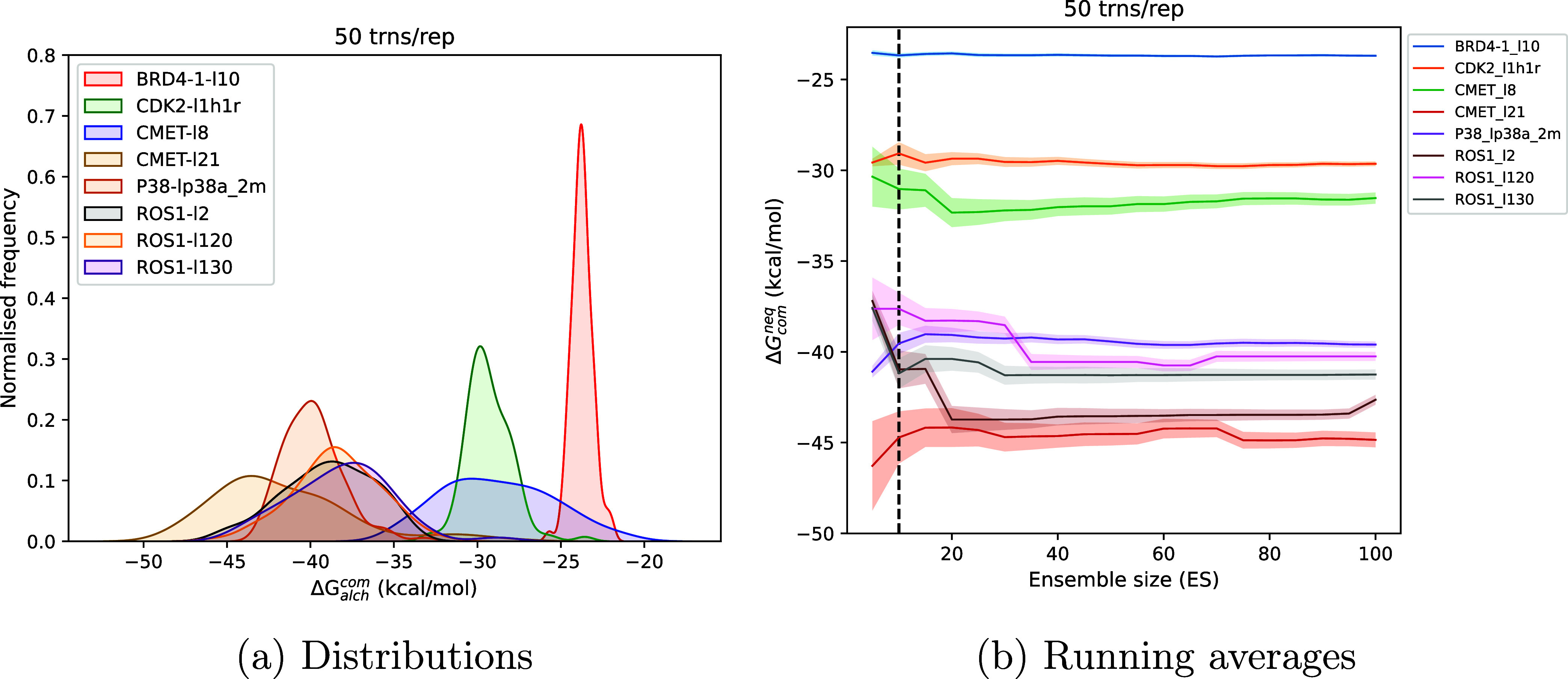
(a) Normalized frequency distributions of predicted ABFEs for the nonequilibrium approach using ensemble size 100 for a subset of ligand-protein complexes depicting their non-Gaussian nature. (b) Running averages of predicted ABFEs varying with the ensemble size. The dashed line denotes ensemble size 10. The results obtained using 50 transitions per replica (trns/rep) have been included in these plots.

We have previously reported that the MD method is intrinsically chaotic, and given that we are calculating equilibrium properties, it is a requirement that the dynamics must be mixing (in the language of ergodic theory).^40^ Therefore, we are obliged to use a probabilistic representation in order to control uncertainties.^1,38,43^ As a result, the distributions of equilibrium properties (such as free energies) obtained from independent MD trajectories differing only in their starting velocities can vary substantially.^41,42,44^ Moreover, the nature of such distributions is generally not normal.^33,39,52^ In this study, we have performed ensemble simulations of large size (whose members contain up to 100 replicas) for a subset of systems using both EQ and NEQ approaches. Figure 6a displays distributions of Δ*G*_alch_^com^ obtained from each replica individually for the EQ approach. Figures 7a and S8(a,c) capture the corresponding display of data for the NEQ approach but using a different subset of ligand-protein complexes. It should be noted that the results displayed in Figure 7a were obtained using 50 transitions per replica. Similar distributions and running averages using 10 and 100 transitions per replica are included in the Supporting Information (Figure S8(a,c)). It is evident that the distributions deviate from Gaussian behavior in many cases. Note that the widths of these distributions vary by over 30 kcal/mol in numerous cases. This in turn implies large uncertainty, especially if one seeks to use Δ*G* values to select the best ligands in a drug discovery context. Figure S13 displays the effect of transition lengths (1, 2, and 4 ns) on Δ*G*_com_ distributions; it is evident that transition lengths do not affect the width or nature of these distributions. Figure S13 also exhibits non-normality in Δ*G*_com_ distributions. We recently reported similar non-normal distributions for experimental binding affinities too.^79^

Table 2 shows the skewness and excess kurtosis coefficients for each of the distributions included in Figures 6 and 7. It is clear that the majority of systems have positive excess kurtosis coefficients going up to as high as 3.78. This implies that the tails of the corresponding distributions are fat and there is a significant probability of obtaining many different Δ*G* values. It should be noted that we also need large ensembles to detect non-normality^81,82^ and hence performing a handful of “repeats” would never capture the possible multimodal features of a given system.

**Table 2:** Skewness and Excess Kurtosis Coefficients for the Systems Studied Using Ensemble Size 100

approach	protein	ligand	skew	kurt
EQ	BRD2_2	Bromosporine	0.70	1.92
	BRD3_1	RVX-208	0.32	–0.44
	BRD4_1	l6	0.18	–0.08
	BRDT_1	RVX–OH	–0.29	0.13
	CDK2	l20	–0.03	0.31
	CMET	lCHEMBL3402745	–0.18	0.31
	Galectin	lF	0.35	0.28
	JNK1	l17124–1	0.55	0.54
	P38	lp38a_2m	–0.18	0.06
	PDE2	l43249674	0.09	–0.25
	TYK2	lejm_48	0.16	0.08
				
NEQ	BRD4_1	l10	–0.01	0.12
	CDK2	l1h1r	0.35	0.36
	CMET	lCHEMBL3402761_1_21	0.81	0.11
	CMET	lCHEMBL3402748_5300_8	0.24	–0.38
	P38	lp38a_2m	1.15	3.78
	ROS1	l2	–0.46	–0.27
	ROS1	l120	0.44	0.15
	ROS1	l130	0.42	0.27

### 3.2 Overlap between Work Distributions

In case of the NEQ method, it is necessary (but not sufficient) for the distributions of work values in forward and reverse directions to have non-negligible overlap with each other in order to obtain reliable ABFE predictions.^15,83^ In cases of poor or no overlap, the predictions are at best biased but most likely incorrect, hence highly unreliable. Thus, it is important to check the extent of overlap between the two distributions and ensure that it is nonzero. None of the published articles reporting the applications of NEQ approaches for ABFE predictions even discuss this issue, let alone quantify the extent of such overlap. In the case of RBFEs, Procacci discussed this matter briefly,^84^ and more recently, we discussed it in greater detail.^16^ For ABFEs, Gapsys et al.^21^ suggested a metric to check the overlap but used only a small data set of 7 protein–ligand systems. Here, we define a quantity termed the “overlap coefficient” which is defined as the area of intersection of the two work distributions. A value of zero indicates that there is no overlap between the two distributions, whereas a value of 1 denotes identical distributions in both directions. We calculate overlap coefficients for each ABFE prediction in this study and systematically investigate the factors affecting its value.

**Figure 8:**
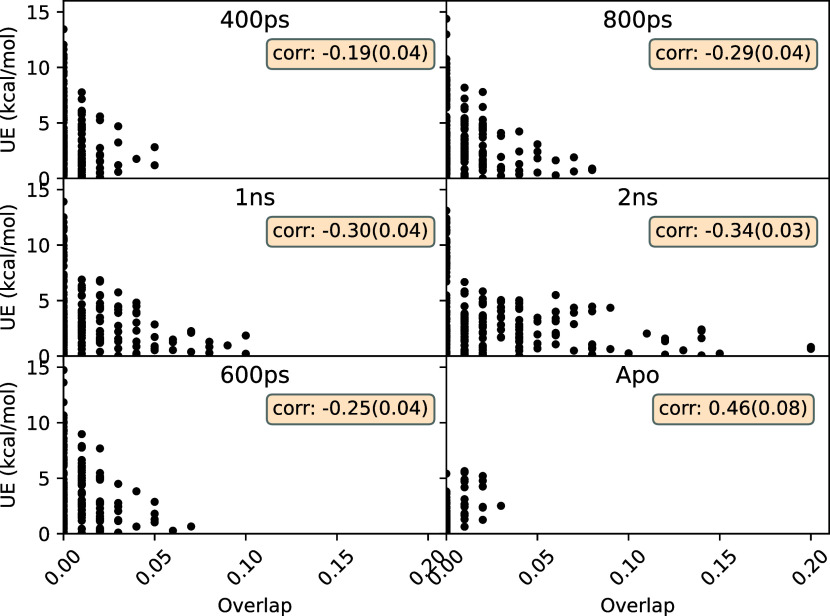
Correlations between unsigned error (UE) and overlap coefficient for various transition lengths in case of the NEQ method. “600 ps” denotes the recommended standard protocol, whereas “Apo” denotes the same protocol but using *apo* structures at λ = 0 end-point. Pearson’s *r* is shown in the inset. Weak anticorrelations in most cases emphasize the necessity of nonzero overlap for unbiased ABFE predictions. The majority of cases with large UEs have zero overlap.

Table S3 contains the range of overlap coefficients for each system separately as well as the overall data set using different durations of the fast alchemical transition − 400 ps, 600 ps (our standard protocol), 800 ps, 1 ns, and 2 ns. It also includes the number of complexes with zero overlap for each case. Strikingly, the number of complexes with zero overlap between the two work distributions are 125 (57.08%), 108 (49.32%), 99 (45.21%), 87 (39.73%), and 60 (27.40%), respectively, for 400 ps, 600 ps, 800 ps, 1 ns, and 2 ns long transitions. The corresponding ranges of overlap coefficients are 0.00–0.05, 0.00–0.07, 0.00–0.08, 0.00–0.10, and 0.00–0.20. It is clear that an increase in transition length leads to better overlap which is expected due to smaller work dissipation, in turn leading to work values closer to each other in the opposite directions (also see Figure S2). However, a significant fraction of complexes have zero overlap in all cases, going down to only about 27% even when using a 2 ns long transition, a value much higher than the transition length generally used in the literature. This doubtlessly contributes to larger uncertainties observed in general with the NEQ approach as compared to the EQ approach. Further, Figure 8 displays the correlation between unsigned error (UE) and overlap coefficient for the entire data set using the same transition lengths. We can see weak anticorrelation between these quantities in all cases which is expected given the necessity of nonzero overlap for unbiased estimates. More importantly, the majority of complexes with large UE are the ones that have zero or very small overlap coefficients. It should be noted that these values are obtained using an ensemble size of 10, replica length of 10 ns and 500 transitions at each end-point making our findings statistically robust.

**Figure 9:**
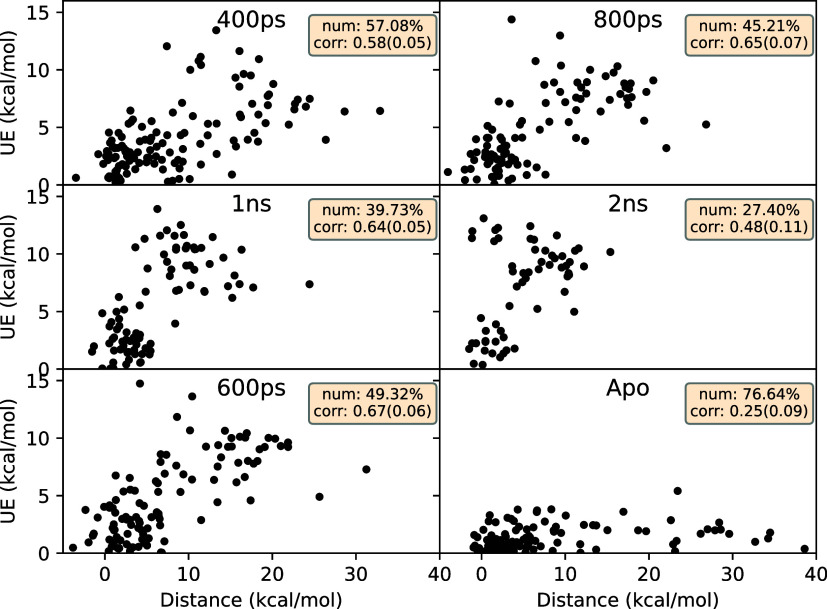
Correlations between unsigned error (UE) and distance values for various transition lengths in case of the NEQ method. “600 ps” denotes the recommended standard protocol, whereas “Apo” denotes the same protocol but using *apo* structures at λ = 0 end-point. “num” and “corr” in the inset denote the fraction of total data set (in % terms) with zero overlap and Pearson’s *r*, respectively, in each case.

Overlap coefficients furnish an incomplete picture as they do not quantify the actual distances between the two distributions. In addition, they do not always capture the existence of outliers in one or both distributions. It may be possible that, despite a zero overlap, the distributions are very close to each other and hence the predicted Δ*G* may not be too biased. Therefore, we define another metric named “distance” which is the difference between the inner extremes of the two work distributions. In other words, it is the difference between the smallest value of the distribution toward the right (forward transition) and the largest value of the distribution toward the left (reverse transition). Figure 9 displays the correlation between UE and distance for all complexes with zero overlap. It is clear that UE and distance correlate well, providing clear evidence that NEQ calculations are inaccurate if the work distributions are not overlapping, and become increasingly so as they move further apart.

We note that there is a handful of cases with negative distance values despite zero overlap. These correspond to situations where both distributions have such outliers that fall within the range of values (extended due to the presence of outlier(s)) of the other distribution. There may also be cases where outliers exist in one of the distributions leading to artificially reduced distance values. All these factors reduce the correlation between UE and distances and can only be addressed properly on performing ensemble simulations.

It is clear from Figure 9 that distances decrease with increasing transition lengths. We further investigated the effect of two other NEQ parameters on this phenomenon. Figure S14 displays the dependence of distances on the ensemble size of the end-point simulations as well as the number of transitions per end-point replica (denoted as “trns/rep”) for a subset of systems used for extended studies. As expected, we see that distance values reduce on increasing ensemble size as well as trns/rep. However, the most important thing to note from Figure S14 and Table S3 is that out of all three NEQ parameters, distance values are the most sensitive to trns/rep. With the same amount of enhancement in any one of the parameters, trns/rep leads to the highest reduction in distances. Most striking from Figure S14 is the fact that there may be cases where distances do not diminish to zero even on increasing trns/rep, ensemble size and transition length to 100, 100, and 2 ns, respectively. This is a major limitation of the NEQ approach, not to mention the high computational costs associated with such parameter adjustments.

### 3.3 Factors Affecting Predicted Free Energies

Capitalizing on the large size of our data set, we discuss in detail and in a systematic way the various factors that affect the reliability of ABFE predictions obtained using either approach. Some factors are common between both EQ and NEQ approaches, whereas others are specific to the NEQ approach. We would like to clarify here that there is one parameter specific to the EQ approach—the set of λ-intermediates—that is kept fixed in this study (refer to Section 2.2 for details). We acknowledge that it is possible to choose the intermediates adaptively for each system for optimized efficiency as shown in our previous studies.^85−87^ However, this is out of the scope of our study so we choose a set that is expected to be suitable for most systems. Δ*G*_com_ was used to assess the variance/convergence of our calculations as this component dominates the overall variance/convergence of the final predicted BFE.

#### 3.3.1 Ensemble Size

The previous discussion on the non-normality of ABFE distributions using an ensemble size of 100 for a subset of systems provides categorical evidence that predicting ABFEs from one-off simulations is highly unreliable. We extend that analysis here by using the entire data set of 219 protein–ligand complexes for which we have used an ensemble size of 10. Table 3 includes the variation of MUE, RMSE, Pearson’s *r*, and Spearman’s ρ across replicas for the EQ approach. In other words, it includes those values obtained for the full data set of 219 complexes on using only a single replica (denoted by “Replica ID”) out of the ensemble of 10 for each complex. This gives ten different MUEs, RMSEs, *r*, and ρ values for the full data set ranging between 1.16 and 1.38 kcal/mol, 1.54–1.81 kcal/mol, 0.61–0.70, and 0.63–0.71, respectively. The table also includes values of all these statistical measures when ABFEs are obtained using ensemble sizes 3, 5, 8, and 10 (denoted as “ES3–10”) for both approaches. In case of the NEQ approach, the results were obtained using 50 transitions per replica in each direction. Using the EQ approach, MUE, RMSE, *r*, and ρ using ES3 are 1.09(0.07) kcal/mol, 1.46(0.08) kcal/mol, 0.72(0.03), and 0.71(0.04), respectively, which are all better than the corresponding best values using any single replica. Increasing the ensemble size from 3 to 10 leads to a steady improvement in these metrics with ES10 yielding corresponding values of 0.93(0.05) kcal/mol, 1.22(0.06) kcal/mol, 0.77(0.03), and 0.76(0.04), respectively. The same is true for the NEQ approach as well, with MUE, RMSE, *r*, ρ values improving from 1.39(0.08) kcal/mol, 1.80(0.09) kcal/mol, 0.63(0.04), 0.61(0.05) for ES3 to 1.15(0.07) kcal/mol, 1.52(0.09) kcal/mol, 0.70(0.04), 0.68(0.04) for ES10 respectively. This further indicates that the ensemble based approach makes results more reliable irrespective of the method used.

**Table 3:** Variation of Overall Accuracy across Replicas and the Effect of Ensemble Simulations^a^

	replica ID or	all					
approach	ensemble size (ES)	MUE	RMSE	*r*	ρ	σ > 0.5	Ovlp = 0
EQ	1	1.34(0.07)	1.73(0.09)	0.64(0.04)	0.64(0.04)		
	2	1.34(0.08)	1.81(0.11)	0.64(0.04)	0.63(0.05)		
	3	1.31(0.07)	1.69(0.09)	0.68(0.03)	0.66(0.04)		
	4	1.32(0.07)	1.70(0.10)	0.69(0.04)	0.70(0.04)		
	5	1.24(0.07)	1.60(0.08)	0.66(0.04)	0.64(0.05)		
	6	1.18(0.07)	1.54(0.09)	0.69(0.03)	0.68(0.04)		
	7	1.34(0.07)	1.65(0.07)	0.66(0.04)	0.65(0.04)		
	8	1.16(0.07)	1.54(0.09)	0.70(0.03)	0.71(0.04)		
	9	1.38(0.07)	1.71(0.08)	0.68(0.04)	0.66(0.05)		
	10	1.23(0.08)	1.70(0.14)	0.61(0.06)	0.64(0.05)		
	ES3	1.09(0.07)	1.46(0.08)	0.72(0.03)	0.71(0.04)	42.92	
	ES5	1.01(0.06)	1.33(0.07)	0.75(0.03)	0.74(0.04)	27.85	
	ES8	0.95(0.05)	1.23(0.07)	0.77(0.03)	0.76(0.03)	9.59	
	ES10	0.93(0.05)	1.22(0.06)	0.77(0.03)	0.76(0.04)	5.02	
							
NEQ	ES3	1.39(0.08)	1.80(0.09)	0.63(0.04)	0.61(0.05)	68.49	72.15
	ES5	1.30(0.08)	1.75(0.11)	0.64(0.04)	0.64(0.05)	66.21	63.01
	ES8	1.19(0.08)	1.63(0.11)	0.68(0.04)	0.68(0.04)	59.82	52.97
	ES10	1.15(0.07)	1.52(0.09)	0.70(0.04)	0.68(0.04)	53.88	49.32

a “σ > 0.5” and “Ovlp” correspond to the fraction of complexes (in % terms) with error bars greater than 0.5 and zero overlap coefficients respectively. All energies are in kcal/mol.

The second last column of Table 3 contains the fraction of complexes (in % terms) with error bars (σ) greater than 0.5 kcal/mol which is 43%/68% in case of ES3, and reduces to 5%/54% in case of ES10 with intermediate values for ES5 and ES8 for EQ/NEQ. The last column contains the fraction of complexes (in % terms) with zero overlap (in case of NEQ) that reduces from 72% for ES3 to 49% for ES10. All this conclusively demonstrates that both accuracy and precision improve substantially with increasing ensemble size and that reporting results from a single replica is not reliable. We note that ensemble simulations based only on varying initial conditions address the aleatoric uncertainty but cannot eradicate any systematic bias prevalent in the system due to factors such as force field inaccuracies, errors in experimental data and so on. This is generally true for any enhanced sampling method, as also reported in the case of REST2 in previous studies.^33,34,36^ The bias due to differences in conformations (such as binding poses) may be overcome by performing ensemble simulations with varying initial structures. The other factors responsible for the systematic bias are captured by the uncertainties in the epistemic parameters, namely those which can be improved by gathering more data and/or improving the theoretical understanding of the system/method. Ensemble simulations can be used to control epistemic uncertainty in order to alleviate systematic bias. It is a major undertaking and computationally demanding which is out of the scope of the current study. However, in the present study, all such biases are encompassed in the empirical constant proposed here (see the Supporting Information for details). Vassaux et al.^38^ and Edeling et al.^1^ show how to use ensembles to control epistemic uncertainties.

Figures 6b and 7b display the variation of Δ*G*_com_ with ensemble size increased up to 100 for a subset of systems using EQ and NEQ approaches. The dashed line denotes ensemble size 10. As can be seen very clearly, Δ*G*_com_ converges in most cases after ensemble size 10, before which fluctuations could be observed. There are a few exceptional cases like ROS1 when employing the NEQ approach where there are several other adjustable parameters as discussed below. Therefore, we recommend using an ensemble size of 10 in general with a possibility of further increase if and when required; for instance, cases where non-normality is of interest and/or when error bars are above the desired value.

#### 3.3.2 Replica Length

The duration of such simulations is an obvious quantity that affects the predicted ABFEs. To systematically study its effect on the accuracy and precision of our predictions, we extended the length of each replica within the ensemble-simulation to 100 ns for a subset of 73 complexes (for the EQ approach) and to 50 ns for a subset of 81 complexes (for the NEQ approach). Table 4 compares RMSE values as well as Δ*G*_conf_^prot^ between replica lengths 10 ns and 100/50 ns for all these complexes system-wise. Figure S10 displays a comparison between results from the first and the last 10 ns of the total 50 ns long replicas in case of NEQ calculations for 81 complexes systemwise. It is apparent that the effect of replica length is system-dependent. However, a closer look at the data reveals some interesting patterns. In all systems, the extent of change in raw RMSE and Δ*G*_conf_^prot^ values (between 10 ns and 50/100 ns) are correlated such that a higher/lower change in raw RMSE is accompanied by a change of similar magnitude in Δ*G*_conf_^prot^. This is true for both EQ and NEQ approaches. This means that with longer simulation duration, conformational sampling of both *holo* and *apo* states may take place, improving the overall accuracy of predictions. However, this phenomenon is system-dependent and may not happen at all or only to a small extent for some systems even at 100/50 ns (such as Bromosporine, CDK2, TYK2, BRD4–2). This is evident from the large differences between RMSEs before and after adjustment for various systems using the replica length of 100 ns (2.26 kcal/mol for CDK2 and 1.06 kcal/mol for bromosporine) in case of the EQ approach and 50 ns (2.61 kcal/mol for BRD4–2, 6.61 kcal/mol for ROS1, 6.41 kcal/mol for TRKA and 6.71 kcal/mol for TYK2) in case of the NEQ approach. This is in agreement with a previous study where the authors studied two systems using extended simulations (1 μs long) to report that the accuracy of ABFEs improved only for one system, whereas it deteriorated for the other.^31^ Furthermore, RMSEs after adjustment (denoted as “RMSE^adj^”) are very similar at 10 and 100 ns in all cases (the largest difference being 0.43 kcal/mol in case of BRD4–1). This implies that a simple adjustment using an empirical correction term Δ*G*_conf_^prot^ yields the benefits of extended simulation duration in most cases (with the obvious *caveat* that reference data is necessary to perform such a correction). Therefore, it is not desirable to run long duration simulations in general for ABFE calculations if we are only interested in the relative ranking of compounds.

**Table 4:** Comparison of RMSE (before and after Adjustment with Δ*G*_conf_^prot^) between Different Replica Lengths^a^

			10 ns			100/50 ns (EQ/NEQ)		
method	system	# of complexes	RMSE	RMSE^adj^	Δ*G*_conf_^prot^	RMSE	RMSE^adj^	Δ*G*_conf_^prot^
EQ	BRD4–1	8	2.38(0.61)	1.83(0.36)	–1.52(0.64)	1.52(0.38)	1.40(0.22)	–0.58(0.49)
	Bromosporine	22	2.98(0.34)	1.55(0.26)	–2.54(0.33)	3.03(0.53)	1.97(0.42)	–2.31(0.42)
	CDK2	15	3.73(0.22)	1.00(0.18)	3.60(0.26)	3.25(0.23)	0.99(0.18)	3.09(0.25)
	CMET	12	2.95(0.36)	1.37(0.20)	–2.62(0.39)	1.62(0.25)	1.36(0.22)	–0.87(0.39)
	PDE2	16	2.31(0.28)	1.41(0.16)	–1.83(0.35)	1.88(0.26)	1.23(0.15)	1.42(0.31)
NEQ	BRD4–2	9	4.16(0.31)	0.84(0.25)	–4.07(0.84)	3.47(0.33)	0.86(0.29)	–3.36(0.97)
	CDK2	16	1.85(0.17)	0.93(0.16)	1.60(0.93)	1.40(0.18)	0.99(0.16)	0.99(1.07)
	ROS1	24	9.93(0.32)	1.58(0.17)	–9.80(1.58)	8.24(0.34)	1.63(0.20)	–8.08(1.93)
	TRKA	16	10.24(0.37)	1.50(0.20)	–10.13(1.50)	8.03(0.39)	1.62(0.28)	–7.86(2.11)
	TYK2	16	7.68(0.35)	1.25(0.33)	7.58(1.25)	7.95(0.33)	1.24(0.27)	7.86(1.32)

a An ensemble size of 10 has been used for all cases. NEQ results are obtained using 500 transitions in each direction and the transition length of 2 ns. All energies are in kcal/mol.

With regard to the optimal replica length in such calculations, we recommend 10 ns per replica for equilibrium simulations (applicable to both EQ and NEQ approaches) based on our results in Figure S9 where we display the running averages of Δ*G*_com_ for all 73 complexes up to 100 ns replica length for each system separately. It is manifest that in most cases, the Δ*G*_com_ value either converges or changes only very slightly after 10 ns. Nevertheless, we would like to emphasize that all these observations are only relevant when one employs ensemble simulations (all our results in Table 4 and Figures S9 and S10 use an ensemble size 10). It is clear from our analyses in this and the previous section that running 10 replicas of 10 ns duration each is much better than running a single replica of 100 ns duration, fully consistent with our many previous findings.^9,10,16,41^

#### 3.3.3 NEQ-Specific Parameters Choices

The NEQ approach for calculating ABFEs involves some parameters that are specific to this method (referred to as NEQ parameters hereafter). A systematic analysis of the effect of these parameters on the accuracy and reliability of NEQ ABFE predictions is hitherto not available. A few recent studies have used *ad hoc* values for NEQ parameters without providing proper justification. In this study, we have performed such a systematic analysis and discussed how each of the NEQ parameters affect predictions and make recommendations to optimize their values for ABFE calculations. We also previously reported such an analysis for NEQ RBFE calculations.^16^

##### 3.3.3.1 Number of Transitions

The number of fast alchemical transitions between the two physical end-states is an important parameter in the NEQ approach. Such transitions need to be initiated from equilibrium conformations at each end-state and these conformations must be representative of the true conformational landscape in that state. Further, the number of transitions needs to be sufficiently large to ensure that the distributions of work values obtained in the forward and reverse directions overlap. In general, only a single or a handful replicas have been used by most practitioners and around 100 transitions were run in each direction. However, in this study, we perform ensemble-based simulations at each end-point so we can control the number of transitions by changing either the number of transitions per end-point replica or the number of end-point replicas themselves. We systematically investigated both these parameters and compare the results to recommend the optimal choice. For RBFE, we found that at least 100 transitions are required in each direction for reliable results.^16^

**Figure 10:**
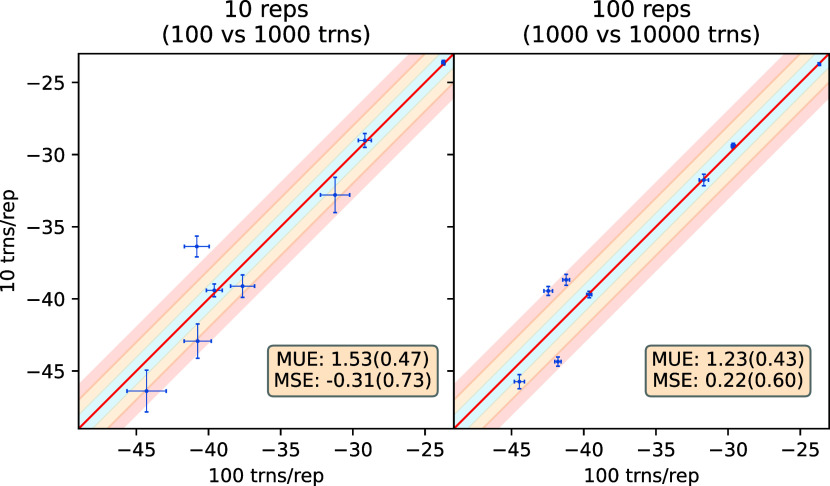
Comparison between ABFE predictions with 10 and 100 NEQ transitions per end-point replica (denoted as “trns/rep”) using 10 replicas and 100 replicas at each end-point. The solid red line denotes the perfect correlation, whereas the blue, orange and red shaded regions represent ±1, ±1–2, and ±2–3 kcal/mol ranges.

We take a subset of 8 different ligand-protein complexes to study the effect of this parameter. Figure 10 compares ABFE predictions obtained with 10 and 100 NEQ transitions per end-point replica (trns/rep) using 10 replicas and 100 replicas at each end-point. Overall, the left panel compares predictions using 100 versus 1000 transitions in each direction whereas the right panel compares those using 1000 versus 10,000 transitions in each direction. It is apparent that the two results agree in some cases but differ in others. Thus, 100 transitions is the minimum requirement, but it is insufficient in the majority of cases. For instance, in Figure 10 only 3/4 cases (out of total 8) fall in the blue shaded region for ensemble size 10/100.

**Figure 11:**
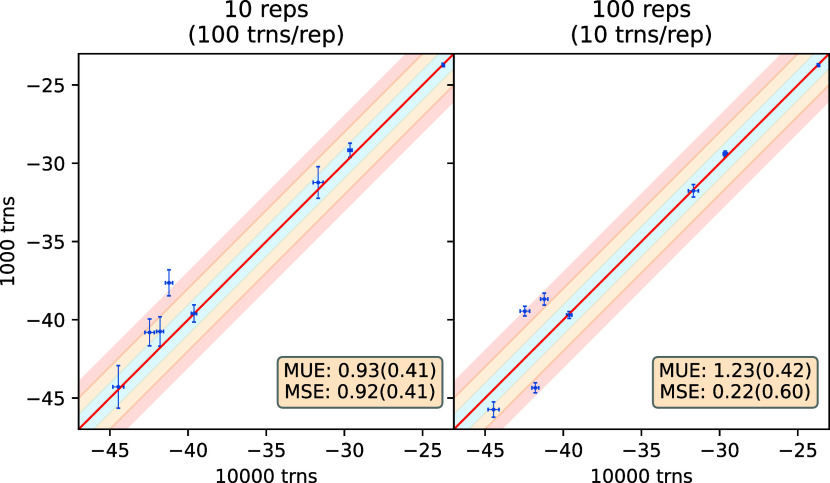
Comparison between ABFE predictions with overall 1000 and 10,000 NEQ transitions (denoted as “trns”) in each direction using 10 replicas (100 trns per replica) and 100 replicas (10 trns per replica). The solid red line denotes the perfect correlation, whereas the blue, orange and red shaded regions represent ±1, ±1–2, and ±2–3 kcal/mol ranges.

But which is the optimal way to increase the number of transitions when required: increasing the ensemble size, the number of transitions per end-point replica or some combination of both? We have obtained ABFEs with 1000 transitions in each direction using two different ensemble sizes: 10 (with 100 trns/rep) and 100 (with 10 trns/rep). Figure S11 compares results using both these settings with each other. The corresponding mean signed and unsigned errors are 0.70(0.48) and 1.04(0.39) kcal/mol respectively indicating that the values do not agree too well, although the larger ensemble has smaller error bars and thus better precision. Next, we compare both settings of overall 1000 transitions with that of 10,000 transitions (100 replicas × 100 transitions per replica, assumed to be the most reliable) in each direction in Figure 11. It is clear that, with smaller MUE, increasing the number of transitions per replica is a preferred way of increasing the overall number of transitions given that a minimum ensemble size is used. This is consistent with our previous observations (Figures 9 and S14) that enhancing trns/rep brings about the highest reduction in distances. Given that 10 trns/rep is found insufficient in the majority of cases, we recommend using 50 trns/rep as it provides results closer to 100 trns/rep in most cases with half the computational cost (see Figure S14). In combination with ensemble size 10 and replica length 10 ns, this leads to overall 500 transitions (chosen uniformly at an interval of 200 ps) for ABFE simulations in the protein environment; whereas for those in the water environment, an ensemble size of 5 should be used leading to overall 250 transitions. As for all other parameters, this number should be flexible and may need to be increased where necessary.

##### 3.3.3.2 Transition Length

Another important parameter specific to the NEQ approach is the length of each nonequilibrium transition. It is generally expected to be of short duration such that the system is driven out of equilibrium. However, it cannot be arbitrarily short as for reliable estimates the NEQ approach requires the work values obtained from such transitions in the forward and reverse directions to be overlapping. If the transition length is too small, the work dissipated would be large and hence end up being very different in the two directions leading to widely varying values and compromising the reliability of predicted free energies. On the other hand, longer transitions make the distributions narrower while bringing them closer to each other and may also decrease the overlap.^16^ Further, they add substantially to the cost of each ABFE calculation. Therefore, we need to identify a “sweet spot” for the transition length. We reported that the minimum transition length in case of RBFE calculations should be 250 ps.^16^ In the literature, transition lengths of around 500 ps have been used for ABFEs without any justification.^31^ Gapsys et al.^21^ attempted to investigate this aspect to arrive at 500 ps but their data set was extremely small (just 7 ligand-protein complexes with a rigid protein) and hence their recommendation lacks statistical robustness. With flexible protein–ligand complexes, it has been reported that even 10 ns transition length can be insufficient for “tricky” cases.^15^ In this study we recommend an optimal value for this parameter based on rigorous and systematic analysis. It is clear that there is no one-size-fit-all solution; rather this parameter needs to be selected flexibly.

We used a subset of 34 ligands binding to P38 to investigate this parameter. We varied the transition lengths for this subset from 100 ps to 2 ns in steps. Table 5 compares the effect of varying the transition length on ABFE predictions. MUE (compared with experiment) corresponding to ABFE predictions using different transition lengths is provided. Further, the fraction of complexes (in % terms) with error bars less than or equal to 0.5 kcal/mol and greater than 1 kcal/mol are included for each case along with ranges of uncertainties. Figure S12 displays the corresponding correlation plots for each case including the fraction of complexes with unsigned error less than 1 kcal/mol in the inset. It should be noted that to obtain this data all other parameters were kept constant (ensemble size 10 (5 for ligand in water), end-point replica length 10 ns, 50 transitions per replica). It is clear that with increasing transition lengths, both accuracy and precision of predicted free energies improves consistently. Figure S13 displays the distribution of Δ*G*_com_ using transition lengths 1, 2, and 4 ns for 9 complexes of the BRD4–2 system. It is evident that, on increasing the transition length from 1 to 2 ns, Δ*G*_com_ becomes more negative, but a further increase to 4 ns does not change Δ*G*_com_ anymore.

**Table 5:** Comparison of Results Using Different Transition Lengths for a Subset of 34 Ligands Binding to P38^a^

Trns_len	MUE	σ ≤ 0.5	σ > 1	range
100 ps	1.37(0.23)	2.94	29.41	0.44–1.73
200 ps	1.27(0.14)	2.94	14.70	0.40–1.26
400 ps	0.93(0.15)	32.35	0	0.30–0.89
800 ps	0.79(0.12)	61.76	0	0.20–0.89
1 ns	0.77(0.11)	79.41	0	0.25–0.64
2 ns	0.68(0.09)	94.12	0	0.16–0.55

a MUE refers to mean unsigned error against experimental binding affinities after adjusting for Δ*G*_conf_^prot^ (kcal/mol). Fractions of complexes (in % terms) with uncertainties ≤0.5 kcal/mol and >1 kcal/mol as well as the range of uncertainties (kcal/mol) are provided for each transition length.

Transition lengths of 100 and 200 ps are extremely unreliable with large MUEs (around 1.3 kcal/mol; less than half the complexes with UE < 1 kcal/mol) and error bars (only around 3% of the complexes with uncertainties ≤0.5 kcal/mol). Further, around 30 and 15% of complexes have error bars greater than 1 kcal/mol using 100 and 200 ps, respectively. Extending the transition length to 400 ps and beyond up to 2 ns, the accuracy and precision of ABFE predictions consistently and substantially improve with MUEs below 1 kcal/mol and zero complexes with error bars above 0.9 kcal/mol in all cases. Based on this analysis, it is clear that the minimum acceptable transition length is 400 ps. However, whether it will suffice or not depends on the system and one must be ready to increase it as and when required. Further, as discussed in detail in the Supporting Information (see Figure S2 and the related section), a higher minimum transition length is necessary for ligand coupling in the protein environment for forward transitions in order to reduce extreme work values and improve overlap between work distributions. Therefore, for such transitions we recommend a minimum transition length of 800 ps.

It should be noted that, in order to extend the transition lengths, one would need to rerun the entire set of transitions from scratch which compounds the computational cost, not to mention the inconvenience. This is the reason why we do not recommend starting with any smaller value of transition length in respective cases as only a small fraction of complexes are expected to meet the threshold criterion (see Discussion for details) using relatively smaller transition lengths; while for the rest it would amount to a waste of computational resources.

We applied the information derived from our above findings to the entire data set of 219 complexes by performing ABFE calculations using four different transition lengths: 400 ps, 800 ps, 1 ns, and 2 ns. Table 6 and Figures S4 and S5 contains the results so obtained. Similar trends follow with consistent improvements in both accuracy and precision of ABFE predictions with increasing transition lengths (lower MUE/RMSE and higher percentage of complexes with error bars below 0.5 kcal/mol). Further, the fraction of complexes with zero overlap coefficients reduces substantially from 57 to 27% on going from 400 ps to 2 ns.

**Table 6:** Comparison of Results Using Different Transition Lengths for the Full Dataset^a^

Trns_len	MUE	RMSE	σ ≤ 0.5	σ > 1	range	zero
400 ps	1.20(0.07)	1.60(0.09)	40.64	9.13	0.20–1.46	57.08
800 ps	1.13(0.07)	1.51(0.10)	47.03	8.22	0.12–1.48	45.21
1 ns	1.09(0.06)	1.39(0.07)	51.14	9.59	0.13–1.54	39.73
2 ns	1.00(0.06)	1.30(0.07)	60.73	7.30	0.10–1.60	27.40

a MUE and RMSE refer to the mean unsigned error and root mean squared error, respectively, against experimental values after adjusting for Δ*G*_conf_^prot^ (kcal/mol). The fraction of complexes (in % terms) with uncertainties ≤0.5 kcal/mol and >1 kcal/mol as well as the range of uncertainties (kcal/mol) are provided. “Zero” denotes the number of complexes (%) with zero overlap coefficients.

### 3.4 Adjustments for Apparent Systematic Errors

A known issue with alchemical ABFE is the occurrence of a systematic error in predicted values. This phenomenon is partly attributed to the conformational differences between the *holo* and *apo* structures of the protein environment which represent the two end-points of the thermodynamic cycle used to calculate ABFE. Such systematic errors are system-dependent; they may be absent in some cases, while occurring to different extents in different systems. Both their signs as well as magnitudes vary across systems as is evident from Figures 3 and 2 and Table S2. Further, they are independent of the approach used as we report their consistency across both EQ and NEQ approaches in our results. These errors heavily compromise the accuracy of ABFE predictions and hence make their direct application difficult.

In this study, we have proposed a way to alleviate this issue in a simple way—by introducing an empirical correction term named the “protein conformational free energy” and denoted as “Δ*G*_conf_^prot^”. However, the problem is that it relies on predetermined reference free energies (in our case, experimental Δ*G*) and cannot be applied prospectively when no reference data is available. Therefore, it is of limited applicability when accurate free energies are desired; ranking can still be obtained correctly, but not the absolute Δ*G*s. On the contrary, if an accurate ABFE prediction is not required, then approximate methods such as ESMACS^56,79,88^ can produce a reliable ranking with substantially less computational effort.

Another possible approach to overcome this issue is to increase/enhance the conformational sampling^89^ that may yield a good mix of both *apo* and *holo* conformations at all intermediate states. In this study, we have shown the effect of extended simulation duration on the systematic errors in ABFEs (Table 4). It is clear from our research that such measures do not guarantee a reduction in Δ*G*_conf_^prot^ and any such effect varies substantially across systems. This is expected as sometimes the energy barriers between *apo* and *holo* states are too high to be overcome with standard MD simulations. Further, a standard simulation length or ensemble size cannot be recommended as each system will have its own requirements, if the errors can be reduced at all. Enhanced sampling techniques may be helpful in some cases but the same issues persist with their application too.

#### 3.4.1 Using *Apo* Structures with NEQ Approach

Another way to alleviate the issue of systematic errors in ABFE predictions that is frequently suggested by proponents of the NEQ approach is using both *holo* and *apo* structures for the protein environment in the ABFE calculation.^31^ This is specific to the NEQ approach as the way ABFE calculations are set up within the NEQ approach, allows one to initiate the fast nonequilibrium transitions from conformations sampled using the *apo* structure for the coupling process whereas those sampled using the *holo* structure can be used for the decoupling process. It has been claimed that this will reduce such systematic errors.^31^ Notwithstanding this fact, no systematic evaluation of this effect has been undertaken. Here, we provide a critical assessment of this implementation. Although it is usually promoted as a major benefit of the NEQ approach over the EQ approach for ABFE calculations, we find the picture to be far more nuanced.

**Figure 12:**
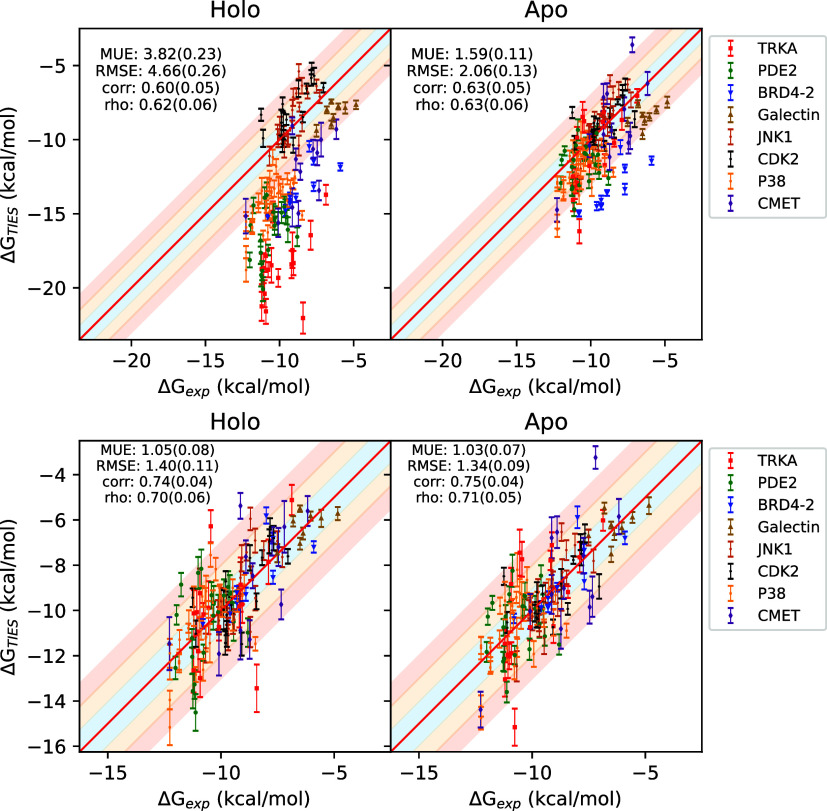
Comparing predictions from the NEQ approach obtained using *holo* structures at both end-points (denoted as *Holo*) against those using *apo* structures at one end-point and *holo* structures at the other end-point (denoted as *Apo*). The top and bottom panels display data before and after adjustment with Δ*G*_conf_^prot^, respectively. A subset of 137 complexes spanning across 8 protein systems have been compared. The solid red line denotes the perfect correlation, whereas the blue, orange and red shaded regions represent ±1, ±1–2, and ±2–3 kcal/mol ranges.

Figure 12 compares Δ*G* predictions using the NEQ approach in two cases: (a) *Holo*—when using *holo* protein structure to generate conformations for initiating transitions at both end-points, and (b) *Apo*—when using *apo* protein structure at the end-point with fully decoupled ligand and *holo* protein structure at the opposite end-point to generate conformations for initiating transitions from respective end-points. The top panel compares the raw data whereas the bottom panel displays the data after adjusting for Δ*G*_conf_^prot^ in each case. As visible clearly in the top panel and also quantified by the corresponding differences in MUE and RMSE values, the systematic errors in the *Apo* case are smaller in general. The same can be inferred from Table 7 that provides more details on this aspect with system-wise comparisons. Four out of the 8 systems compared have substantially smaller Δ*G*_conf_^prot^ values in case of *Apo* scheme as compared to the *Holo* scheme, and the remaining four have no statistically significant differences in Δ*G*_conf_^prot^ between the two cases (with differences ≤0.8 kcal/mol). The latter includes all cases with positive Δ*G*_conf_^prot^ values (CDK2 and JNK1) as they are the ones with no significant conformational differences in *apo* and *holo* states of the protein, as explained in the Supporting Information.

**Table 7:** Comparison of RMSE (before and after Adjustment with Δ*G*_conf_^prot^), Δ*G*_conf_^prot^, and the Percentage of Zero Overlap Cases (“% Zero”) between NEQ Δ*G* Predictions Using *Holo* Structures at Both End-Points (*Holo*) with Those Using *Apo* Structures at One End-Point and *Holo* Structures at the Other End-Point (*Apo*)^a^

		*holo*				*apo*			
system	complexes	RMSE	RMSE^adj^	Δ*G*_conf_^prot^	% zero	RMSE	RMSE^adj^	Δ*G*_conf_^prot^	% zero
BRD4–2	9	4.75(0.29)	0.98(0.25)	–4.65(0.33)	0	4.71(0.26)	0.89(0.26)	–4.63(0.29)	0
CDK2	16	1.61(0.20)	1.17(0.16)	1.12(0.29)	93.75	1.17(0.23)	1.12(0.22)	0.32(0.28)	93.75
CMET	12	4.09(0.46)	1.75(0.33)	–3.70(0.51)	100	1.99(0.28)	1.95(0.32)	–0.36(0.57)	100
Galectin	8	2.02(0.20)	0.60(0.11)	–1.93(0.21)	0	2.22(0.22)	0.71(0.15)	–2.10(0.25)	0
JNK1	21	1.27(0.21)	1.14(0.17)	0.55(0.25)	38.10	1.05(0.16)	0.92(0.15)	0.50(0.20)	38.10
P38	34	3.84(0.22)	1.25(0.17)	–3.64(0.21)	38.24	1.78(0.20)	1.21(0.13)	–1.31(0.20)	100
PDE2	21	5.82(0.36)	1.66(0.23)	–5.58(0.36)	0	1.82(0.24)	1.45(0.17)	–1.10(0.31)	95.24
TRKA	16	8.82(0.50)	1.93(0.46)	–8.60(0.48)	100	2.15(0.38)	1.89(0.32)	–1.02(0.47)	100
All	137	4.66(0.25)	1.40(0.10)	–3.29(0.28)	46.72	2.06(0.13)	1.34(0.09)	–0.96(0.16)	76.64

a All energy values are in kcal/mol. “% zero” refer only to Δ*G*_com_.

The above observations are consistent with a recent report in the literature which sought to establish the superiority of the NEQ approach over other approaches.^31^ Here, we provide a critical aspect of the *Apo* scheme that has not been discussed hitherto in the literature. Looking carefully at Table 7, we find that for 2 out of these 4 systems (P38 and PDE2) where a reduction in Δ*G*_conf_^prot^ is observed, there is also a decrease in overlap between the forward and reverse work distributions (indicated by an increase in the percentage of zero overlap cases); whereas the remaining two (CMET and TRKA) are irrelevant as they have 100% zero overlap using both *Holo* and *Apo* schemes. The number of zero overlap cases increases from 0/38% in the case of the *Holo* scheme to 95/100% in the case of the *Apo* scheme for PDE2/P38. This contributes to the overall increase in zero overlap cases from 47 to 77% for the full data set.

For the remaining four cases (with insignificant changes in Δ*G*_conf_^prot^), the number of complexes with zero overlap coefficients remain unchanged which is an indication of higher similarity between protein structures at the two end-points. This observation is expected as when the *apo* and *holo* protein structures are similar, it is not expected to be associated with a significant change in the value of Δ*G*_conf_^prot^. These provide examples of cases where Δ*G*_conf_^prot^ is a result of factors other than protein conformational changes. Two out of these systems (BRD4–2 and Galectin) have good overlap between forward and reverse work distributions for both *Apo* and *Holo* schemes (no complexes with zero overlap coefficients for both schemes). The other two systems (CDK2 and JNK1—cases with positive Δ*G*_conf_^prot^) have significant fractions (94 and 38% respectively) of zero overlap cases despite similarity between *apo* and *holo* protein structures. This may be caused due to differences in ligand binding poses and/or other factors such as presence of water molecules,^37^ flexible binding pockets and so on. In summary, in cases where the *apo* and *holo* structures differ in a significant way (the only cases where the *Apo* scheme is worthwhile), a reduction of magnitude in Δ*G*_conf_^prot^ is expected, but this comes at a cost of reduced overlap and hence compromised accuracy.

**Figure 13:**
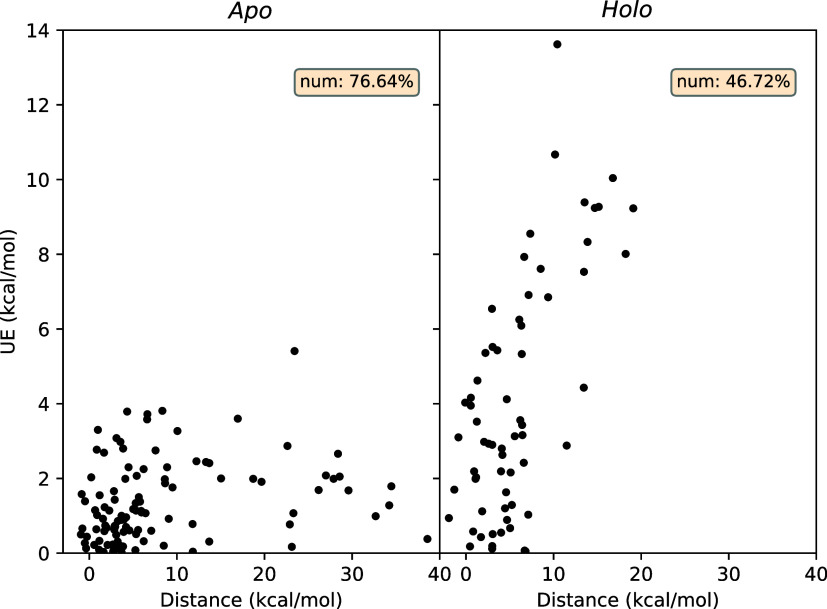
Correlations between unsigned error (UE) using raw Δ*G* values and distance values for *Apo* and *Holo* calculations. “num” in the inset denotes the number of complexes (in % terms) with zero overlap in each case.

To provide a better and more convincing picture, we further investigated this using an analysis of the distance values. Figure 13 displays the correlation between UE and distance for raw Δ*G* values for both *Apo* and *Holo* calculations. It is quite clear that for the *Apo* approach, while the UE values decrease, the distance values increase substantially in comparison to the *Holo* approach. The total number of complexes with zero overlap (included in the inset) is also higher in case of *Apo* as compared to *Holo* (105 vs 64, that is, 77 vs 47%). Out of 137, there are 59 complexes for which the overlap is zero in both *Apo* and *Holo* cases. Figure S15 displays a comparison of the distances for these complexes between *Apo* and *Holo*, showing much larger distances for the former.

The apparent decrease in UE in *Apo* calculations is only a result of a shifted forward work distribution due to different starting conformations for the fast alchemical transition. In the absence of overlap, the predicted Δ*G* using a bidirectional approach (such as Crook’s fluctuation theorem) is essentially an average of the Δ*G* values obtained using the unidirectional approach (such as Jarzynski inequality) in each direction. It is well-known that the latter is highly sensitive to outliers due to exponential averaging and thus biased and unreliable. This is why (a) UE only decreases up to a certain level but not up to the level achieved using the adjustment with Δ*G*_conf_^prot^ (RMSEs: 2.06 vs 1.34 kcal/mol) and (b) it makes the predicted ABFEs biased and unreliable.

When using the *Apo* scheme, additional computational effort is required to achieve nonzero overlaps on a par with the *Holo* scheme, which defeats the whole purpose of using *apo* structures in the first place. Simply asserting that using the *Apo* scheme solves the issue of systematic errors in ABFEs is to the left of disingenuous. The above caveats must be borne in mind while employing this scheme.

Given these constraints, it should be noted that if one is simply interested in ranking a set of ligands and does not bother about the accuracy of absolute Δ*G* values then there is no justification to invest extra computational effort in employing the *Apo* scheme, as is visible from the bottom panel of Figure 12. The accuracies are almost identical for the entire subset of 137 complexes with very similar MUEs and RMSEs whether the *Apo* or the *Holo* scheme is used. In fact, one might argue that approximate ABFE methods such as ESMACS,^11,56^ that provide reliable ranking with much less computation as compared to alchemical ABFE methods, are preferable in such cases.

## 4 Discussion

In this section, we build upon our analyses from previous sections and recommend a definitive protocol to optimally perform ABFE calculations using both EQ and NEQ methods. Further, we also critically compare both methods, highlighting pros and cons of each that should help the community understand which is better.

### 4.1 Recommended ABFE Protocol and Method

In the previous section, we have already recommended optimal values for various parameters involved in ABFE calculations. Here we additionally suggest a “minimum” value for each parameter that may be used in cases where computational resources are limited and/or cost-cutting is desired (for instance, coarse-grained applications at large-scale). Table 8 summarizes all our recommendations. We urge practitioners not to go below the “minimum” value in any case to ensure reliability of results. We acknowledge that there is no one-size-fit-all solution for all systems given the huge variability in protein–ligand structures. Therefore, our recommended protocol is kept flexible such that various parameters will need to be adjusted in order to fulfill two criteria of reliability. The first criterion is bringing the standard errors (using independent estimates from ensemble simulations) below a predefined threshold value. The second one (specific to the NEQ method) is to ensure that the distance value is below a preset threshold value. We suggest using 0.5 kcal/mol as the threshold for standard errors and zero for the distance value which are assumed in our following discussion. However, users may choose their own values based on their requirements, preferences and error tolerance.

**Table 8:** Recommended Values for Various ABFE Parameters^a^

	standard		minimum	
parameter	EQ	NEQ	EQ	NEQ
ensemble size (com)	10	10	5	5
ensemble size (lig)	5	5	5	5
replica length (ns)	10	10	5	5
number of transitions per rep (every 200 ps)	-	50	-	25
Trns length (ps; com in 0 → 1 direction)	-	800	-	800
Trns length (ps; other cases)	-	400	-	400

a Both “Standard” as well as “Minimum” values are included for convenience.

Below we propose the most efficient way to perform ABFE calculations at large scale such that reliability as well as computational efficiency is ensured. First of all, an initial screening of all complexes should be done using our recommended protocol (whether standard or minimum). Thereafter, only the subset of compounds that do not fulfill one or both the above criteria of reliability should be considered for further extended sets of simulations. The extended simulations should be performed in steps, adjusting one parameter at a time in a set order, and filtering out the compounds that fulfill the criteria at each step. This is to ensure that the additional computational costs are minimized.

In the case of the EQ method, ensemble size and/or replica length could be increased to bring uncertainties under the threshold required. We suggest first increasing the ensemble size as uncertainty is better captured by this. In the case of the NEQ method, there is an additional requirement of ensuring overlap and also additional parameters to adjust. Therefore, it is tricky to choose the most efficient order in which to adjust these parameters to ensure reliability. Fortunately, our analyses allow us to provide useful insights in this regard. Given that the distance values are the most sensitive to the number of transitions per replica, we propose to first of all increase its value in a stepwise manner up to maximum 100. This should already filter out a large fraction of the shortlisted compounds. Thereafter, we recommend increasing the transition length up to 2 ns. Finally, for the remaining compounds, ensemble size (followed by replica length) should be increased. The suggested maximum values of 100 transitions per replica (leading to 1000 transitions overall using ensemble size 10) and 2 ns long transitions are provided as guidelines for users to decide when to start adjusting the next parameter in order. However, there may be some cases where one would need to breach even these maximum values depending on system requirements and user preferences.

We compared alchemical ABFE predictions obtained from equilibrium and nonequilibrium approaches. The headline finding is that the results obtained from the EQ approach are both more accurate and precise than those obtained from the NEQ approach. The MUEs for the full data set are 0.93(0.05) and 1.15(0.07) kcal/mol for the EQ and the NEQ approaches, respectively. The corresponding ranges of RMSEs across protein systems for these methods are 0.39–1.83 and 0.60–2.23 kcal/mol and the number of complexes with error bars ≤0.5 kcal/mol are 94.98 and 46.12%, respectively. Moreover, the NEQ approach has more set up parameters to fine-tune which makes it more complicated to implement and automate as well as prone to risk of suboptimal performance. For instance, around 54% of complexes do not fulfill the first criterion of reliability whereas 49% of complexes do not meet the second one when using our recommended standard protocol. Even on extending the transition length to 2 ns, these numbers only go down to 39 and 27% complexes, respectively, for our data set. For the same reason, the NEQ approach exhibits a higher degree of system dependence which makes it largely impossible to apply a standard protocol. Due to these factors, the NEQ approach is plainly less preferable for blind and/or large scale applications.

The proponents of the NEQ method claim that a major advantage of this method over the EQ method is that it is computationally cheaper as well as requiring less wall clock time to run. However, both these claims are unjustified on systematically comparing both methods and implementing them in a reliable and consistent way. In Table 1 and the related section, we have established that the computational costs associated with the NEQ method is only about 10% lower than that of the EQ method using the standard protocol, which is already much less of a saving than often claimed in the literature. Further, the majority of this difference in the overall computational cost is accountable to the thermodynamic leg involving ligand in the water environment. In fact, the simulation time involving protein is 100 ns less in case of the EQ approach (1500 vs 1600 ns; see Table 1) which more than compensates for the higher simulation time required for the ligand-only calculations, effectively nullifying the difference in computational costs of the two approaches. Moreover, our results show that, using the recommended protocols, the number of systems with insufficiently accurate and/or precise results are significantly higher in case of the NEQ approach in comparison to the EQ approach; about half the complexes do not meet both the criteria of reliability as already described above. On top of that, NEQ calculations need to satisfy the additional criterion of nonzero overlap which makes it more stringent. Therefore, the additional simulation time required to be invested in order to get reliable predictions is far higher in case of NEQ, more than wiping out the apparent fractional cost savings. Thus, the real savings in terms of computational costs are negligible at best, if not negative. To make things worse, one cannot reuse the previously run NEQ transitions which need to be rerun from scratch, adding to the computational costs substantially. For instance, simply increasing the transition length alone from 400 to 800 ps adds an extra computation of 800 ns per Δ*G*_com_ component (using the recommended “standard” protocol) since the previous transitions of 400 ps are useless. It should be noted that all the above arguments are equally valid in the case of RBFE calculations using the NEQ approach, due to which the EQ approach is preferable in that case too.^16^

The wall clock time requirements for ABFE calculations are also in favor of EQ in comparison to NEQ. In the EQ approach, the wall clock time required for the entire protocol is the same as that of a single simulation as all replicas can be run concurrently on a large supercomputer. On the other hand, in the NEQ approach, the fast transitions need to be run sequentially after the end-state simulations have completed which adds to the wall clock time required (ignoring the set up and waiting time)—another limiting factor in large-scale applications. For instance, a transition length of 400 ps would require 4% extra time that increases to 10% using 1 ns. Once again, the above is also true in the case of RBFE predictions using the NEQ approach which makes it the less preferable approach.^16^

Finally, we observe that there are systems such as ROS1 for which the standard NEQ protocol does not work. Unlike in the case of EQ, the ROS1 system exhibits substantially larger error bars in the case of NEQ which are related to zero overlap and large distances. For instance, the range of uncertainties for ROS1 system using the NEQ approach is 0.29–1.33 kcal/mol against 0.29–0.59 kcal/mol for the EQ approach. Furthermore, the distance values are nonzero even on increasing the ensemble size to 100, the number of transitions to 10,000 and the transition length to 2 ns (see Figure S14). Overall, it is clear that the EQ method is preferable over the NEQ method for ABFE predictions.

## 5 Conclusions

In this paper, we report on reproducible, accurate and precise ensemble methods for computing alchemical ABFEs and obtain mean unsigned error below 1 kcal/mol. We have assessed both equilibrium and nonequilibrium approaches for a large data set of diverse ligands and proteins. The ensemble EQ approach is found to be more accurate and precise than the ensemble NEQ approach. We have shown that the claims of the NEQ approach being superior to the EQ one simply based on being computationally less expensive are exaggerated and unjustified. Through a comprehensive evidence-based analysis, it has been established that the EQ approach is preferable over the NEQ approach, particularly for large scale applications. In exactly the same way as for RBFE approaches, there are several implementational aspects that render the NEQ approach more complicated, prone to failure and/or unreliable predictions. A systematic investigation of the various parameters involved in ABFE calculations has been performed and based on that, a set of definitive recommendations have been provided (for both EQ and NEQ approaches) that would allow anyone interested to perform ABFE calculations with reliability. In particular, this analysis for the NEQ approach allows practitioners to better understand and control errors, adjusting the large number of parameters involved in this approach. Furthermore, we have provided evidence that coupling two thermodynamic steps (ligand coupling and removal of restraints in the protein environment) in an ABFE calculation into one can reduce the computational costs by 40% without compromising accuracy and/or precision.

Ensemble simulations are necessary to ensure a reproducible method and capture the aleatoric uncertainties associated with the predicted free energies. In future work, they could be used to bring systematic errors arising from the epistemic uncertainties under control. Ensemble methods show that the free energy distributions obtained from independent simulations are non-Gaussian. Moreover, an important requirement for the reliability of NEQ calculations is the overlap between the work distributions in both directions which also requires ensembles. Hitherto completely overlooked, we quantify the overlap requirements and systematically investigate the potential role of various parameters in ensuring that NEQ protocols fulfill these requirements in order to yield reliable results.

Armed with the protocols specified here, one can integrate ensemble alchemical ABFE calculations into large scale drug discovery procedures in an ambitious manner. Using the knowledge gleaned herein on the effect of various parameters and the flexibility of the recommended protocols, it becomes possible to obtain reliable results while optimally utilizing the available computational effort. Importantly, such protocols permit one to perform thousands of calculations concurrently in less than 2 h allowing one to achieve the level of throughput necessary for industrial drug discovery and patient-specific therapeutic applications within the limit of available computational resources.

## Supporting Information Available

Additional data including Tables and Figures (Tables S1–S3, Figures S1–S15); occurrence of extreme work values in the forward direction; coupling of interactions and removal of restraints; origin of the systematic errors (PDF)
