# Supplementary material for: Equilibrium and Nonequilibrium Ensemble Methods for Accurate, Precise and Reproducible Absolute Binding Free Energy Calculations
Source: J Chem Theory Comput. 2024 Dec 16;21(1):440–62. doi: 10.1021/acs.jctc.4c01389 (PMC11736689; doi:10.1021/acs.jctc.4c01389)
Supplement: Supplementary file 1 — ct4c01389_si_001.pdf [file ct4c01389_si_001.pdf]

Supporting Information: Equilibrium and Non-equilibrium Ensemble Methods for Accurate, Precise and Reproducible Absolute Binding Free Energy Calculations

Agastya P. Bhati,[†,§] Shunzhou Wan,[†,§] and Peter V. Coveney[*,†,‡,¶]

†*Centre for Computational Science, Department of Chemistry, University College London, London, United Kingdom*

‡*Computational Science Laboratory, Institute for Informatics, Faculty of Science, University of Amsterdam, Amsterdam,The Netherlands*

¶*Advanced Research Computing Centre, University College London, London, United Kingdom*

§*These authors contributed equally to this study.*

E-mail: p.v.coveney@ucl.ac.uk

Phone: +44 (0)20 7679 4560

Here, we include the supporting information for this study. It covers several important aspects of our findings in more detail, including tables and figures capturing more extensive analyses. These have been referred to at appropriate places in the main text and discussed therein.

Occurrence of Extreme Work Values in the Forward Direction

In this study, we observe a phenomenon specific to the non-equilibrium (NEQ) approach to alchemical ABFE calculation that undermines its reliability. When the ligand is coupled in the protein environment, that is, transformed in the forward direction (corresponding to λ: $0 \rightarrow 1$), the work values obtained are higher than those obtained in the reverse direction (corresponding to λ: $1 \rightarrow 0$), and sometimes can have extreme positive values (going up to 3000 kcal/mol). Table S1 contains frequency distributions of forward work values > 100 kcal/mol for the full dataset (219 complexes) as well as the ROS1 system (24 complexes). There are non-negligible number of work values that are extremely high (> 1000 kcal/mol) with a handul lying in the range of 100-215 kcal/mol. As shown in Table S1, the majority of these extreme values arise from the ROS1 system. Further, the frequency of such extreme values decreases with increasing transition length; going from 343 (0.31%) in case of 400 ps down to 60 (0.05%) in case of 2 ns for the full dataset, and from 286 (2.38%) to 55 (0.46%) for the ROS1 system. Such extreme values degrade both accuracy and precision of NEQ ABFE predictions. It is worth mentioning here that the accuracy remains unaffected by a small fraction of such extreme values when ensemble simulations are employed.

Table S1: Frequency distributions of forward work values > 100 kcal/mol for the full dataset ("Full"; out of total 109500 values) and just the ROS1 complexes ("ROS1"; out of total 12000 values) with different alchemical NEQ transition lengths using three bins (100-215 kcal/mol, 215-1000 kcal/mol and > 1000 kcal/mol). The frequencies in % terms have also been reported for the last bin (> 1000 kcal/mol). The last column contains the largest forward work value from the respective distribution (denoted as W_f^m).

Trns length	Full				ROS1			
	100-215	215-1000	> 1000	W_f^m	100-215	215-1000	> 1000	W_f^m
400 ps	36	0	343 (0.31%)	3152	34	0	286 (2.38%)	3152
800 ps	8	0	151 (0.14%)	2961	8	0	145 (1.21%)	2961
1 ns	7	0	150 (0.14%)	1864	7	0	137 (1.14%)	1864
2 ns	1	0	60 (0.05%)	1777	1	0	55 (0.46%)	1777

The underlying reason for such extreme forward work values is the close contacts between ligand and protein atoms in the starting conformations of these transitions. Such starting conformations are generated using equilibrium simulations with the "ghost" ligand molecule restrained within the binding pocket of the protein. Thus, it is not surprising to have close contacts in such equilibrium conformations. However, when the ligand is coupled by quickly changing λ during fast alchemical transitions, it leads to steric clashes resulting in large values of energy derivatives ($\partial H/\partial\lambda$) for small values of λ. In most cases, such clashes get removed during the course of alchemical transitions; the initially high values of energy derivatives explain the less negative or positive forward work values as compared to reverse work values (see Figure S2). However, in some cases the close contacts in starting conformations cannot be overcome even when the ligand is fully coupled with the environment leading to extreme steric clashes and consequently extreme work values. An example of such a clash is when the protein backbone passes through the center of a ring in the ligand structure. Figure S1(a-b) displays two such starting conformations that led to extreme work values in our simulations. There is no way to avoid these extreme work values in NEQ ABFE calculations as close contacts are bound to occur when the ligand is fully decoupled. One can either ignore the extreme work values (as we have done in this study) and/or filter out starting structures that can possibly lead to such values before even performing the NEQ transition (it would be very difficult to ensure the latter automatically at large scale). In any case, this is an additional drawback of the NEQ approach, particularly when large scale deployment is desired.

As mentioned already, the majority of extreme work values occur in ROS1 complexes. In order to understand the much higher probability of steric clashes in this system, we investigated the structure of its ligands. Figure S1c shows a typical ROS1 ligand belonging to a cogeneric series. ROS1 ligands are large and flexible with several rings and rotatable bonds. The single and double rings at the bottom form the scaffold of this ligand series which is deep in the binding site. The rest of the ligand, including the double ring at the centre and the ring at the top, is half exposed to solvent and flexible. This part of the ligand can

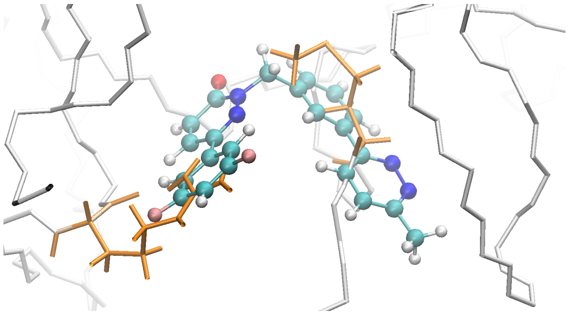

(a) CMET-lCHEMBL3402754_40_14

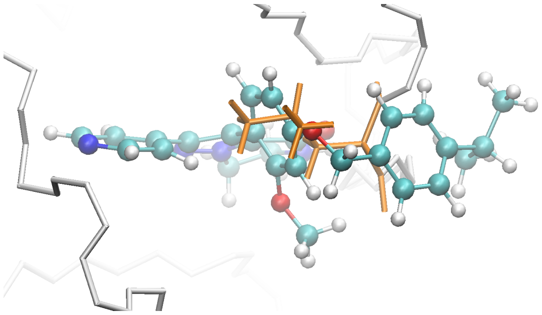

(b) ROS1-l169

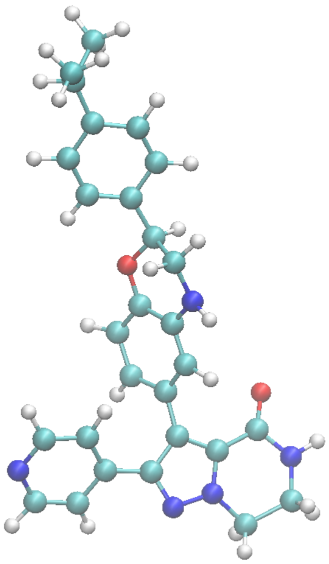

(c) ROS-l169

Figure S1: (a-b) Example starting conformations leading to extreme forward work values in fast alchemical transitions for NEQ calculations. Protein backbone is shown in bond presentation in white and ligands in ball-and-stick presentation. The protein residues having worst conflicts with ligand atoms are highlighted in orange. (c) An example ROS1 ligand displaying its flexible structure with multiple rings. Such structures are prone to prominent steric clashes leading to extreme work values.

have unfavourable contacts with the flexible P-loop of the kinase when interactions between the ligand and its environment are weak or absent. For instance, one of the residues from this loop clashes with the middle part of the ligand as shown in Figure S1b. This explains the higher occurrence of extreme work values in this system and also higher uncertainties in its ABFE preditions (6 out of the total 16 complexes with uncertainties $>$ 1 kcal/mol in the full dataset belong to the ROS1 system).

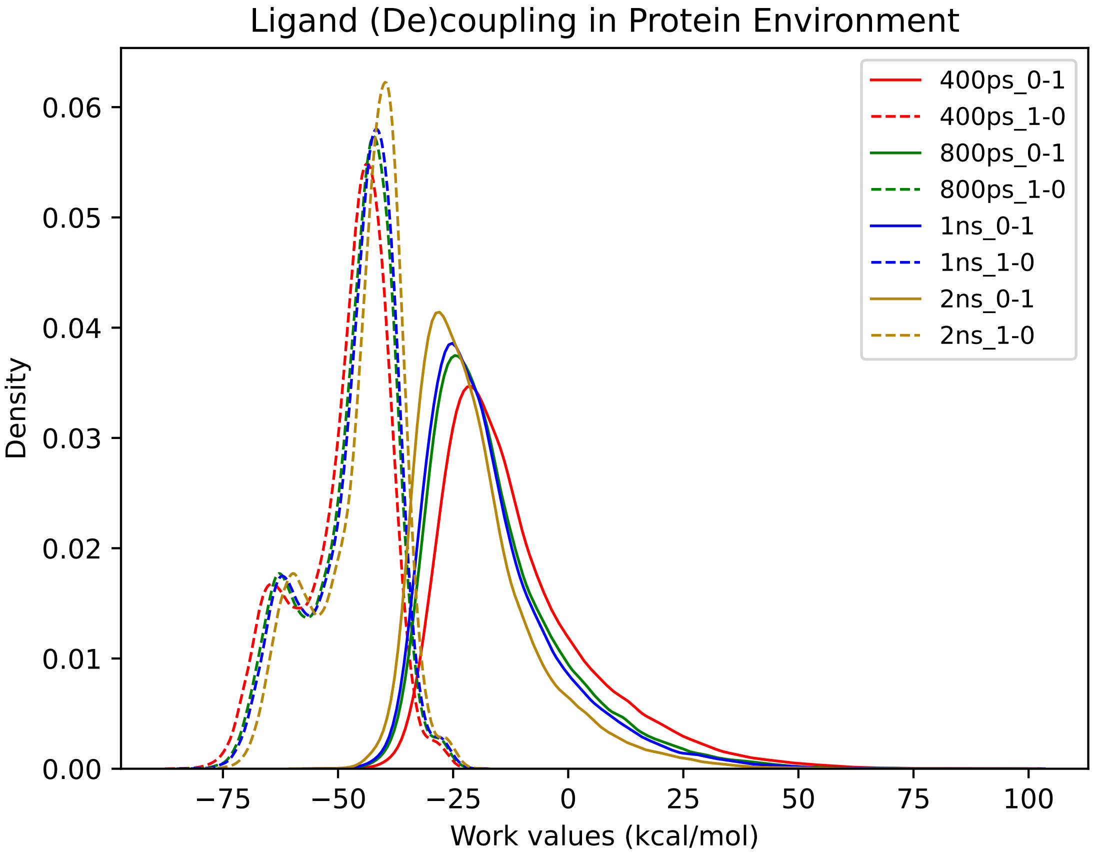

Figure S2: Distributions of NEQ work values for ligand (de)coupling in the protein environment using different transition lengths for the full dataset. The forward ($0 \rightarrow 1$) and reverse ($1 \rightarrow 0$) directions are denoted by solid and dashed lines respectively. The total number of data points in each density plot is 109500 (219 complexes $\times$ 10 replicas $\times$ 50 transitions per replica) less all work values $>$ 100 kcal/mol which have been ignored for better visibility of distributions.

Figure S2 displays the density plots of forward and reverse work values $\leq$ 100 kcal/mol for the full dataset using different transition lengths. It is clear from the figure that: (a) the

distributions of forward work values exhibit heavy tails on the right side; (b) forward work values become increasingly more negative (or less positive) with longer transition lengths and the opposite is true for the reverse work values; (c) the forward and reverse work values get closer to each other with increasing transition lengths (leading to higher probability of overlap between them as also evident from Table S3); (d) however, the shift/change in the reverse work distributions on increasing transition length is smaller as compared the forward work distributions. The last observation is particularly important as it indicates that forward work values are more sensitive to the transition length as compared to reverse work values. In other words, the majority of improvement (in accuracy, precision and reliability) in NEQ resulting from increasing the transition length identically in both directions can be gained by doing so just in the forward direction with half the computational cost. Thus, in order to achieve maximal efficiency, different transition lengths should be used in both directions such that longer transitions are run in the forward direction. This forms the basis of our recommendation of higher transition length in the forward direction as discussed in the main text.

Coupling of Interactions and Removal of Restraints

As discussed in the Methods section, the overall ΔG value is obtained by going through a thermodynamic cycle (Figure 1) consisting of several steps. Two steps involve the ligand in the protein environment: (a) alchemical decoupling of the ligand, and (b) removal of restraints from the fully interacting ligand. Generally, both these steps are performed sequentially increasing the number of intermediate states involved. For instance, when we performed these two steps separately, we used 13 intermediate states for the alchemical step and 12 intermediate states for the restraining step. Overall, this led to 25 intermediate states with an ensemble of simulations performed at each. The ΔG_{com} values so obtained are denoted as "ΔG_{com}^{25}".

We synchronise both these steps such that a single transformation is able to capture

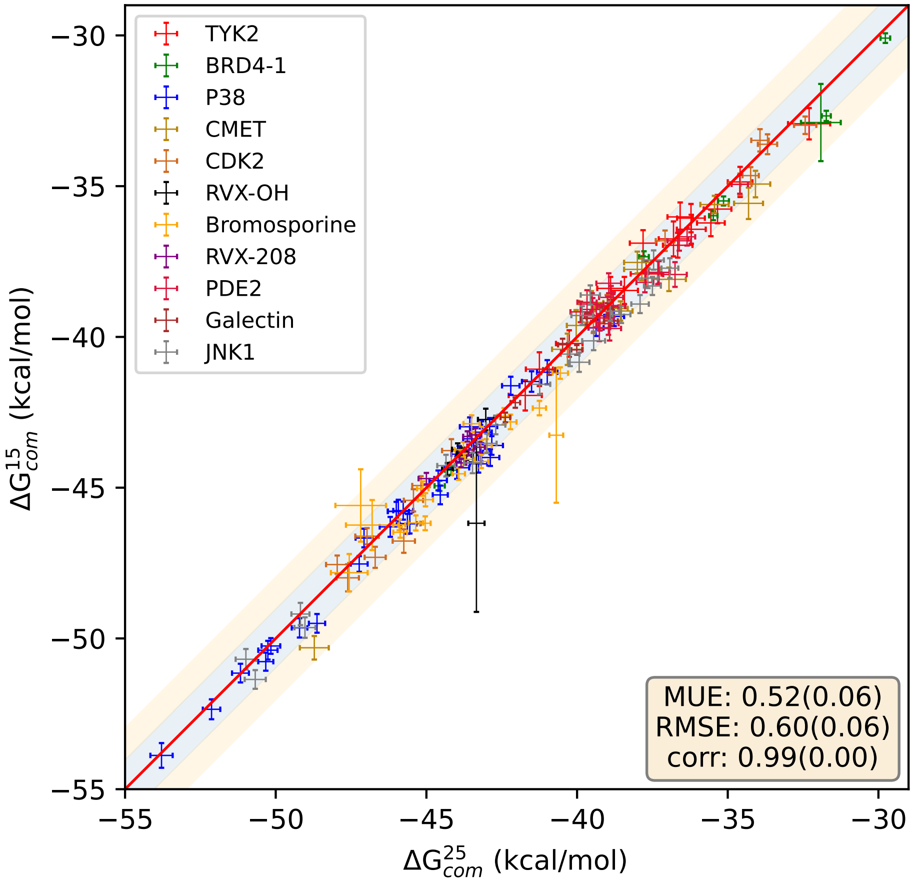

Figure S3: Correlation between ΔG_{com} obtained from the two approaches of either simultaneously (15 intermediate states) or separately (25 intermediate states) performing the alchemical step and the restraining step of the ABFE thermodynamic cycle. The solid red line denotes perfect correlation, whereas the blue and orange shaded regions represent ± 1 and $\pm$ 1-2 kcal/mol ranges.

both these changes simultaneously with the two end-points defined as fully interacting unrestrained ligand and fully restrained non-interacting ligand. In other words, as the strength of a ligand's interactions with its environment is increased, the strength of restraints is simultaneously decreased and vice-versa. In this study, we used 15 intermediate states to fully perform this transformation and the corresponding ΔG_{com} is denoted as "ΔG^{15}_{com}". We found that both these approaches agree very well. We compared both approaches on a subset of 169 complexes with RMSE and Pearson's r of 0.60(0.06) kcal/mol and 0.99(0.00) respectively. Figure S3 displays the near perfect correlation between ΔG^{15}_{com} and ΔG^{25}_{com}. This finding

is relevant as it shows that we are able to obtain almost identical results at only 60% the computational cost — a major factor for large-scale applications.

Origin of the Systematic Errors

In this section, we briefly explain the underlying causes for the observed systematic errors and their implications. The empirical constant capturing such errors is given by the difference between the predicted and the experimental (or a reference) value (see Equation 1).

$$
\begin{aligned}
E =& \Delta G_{calc} - \Delta G_{exp} \\
=& (G^{holo}_{calc} - G^{apo}_{calc} - G^{lig}_{calc}) - (G^{holo}_{exp} - G^{apo}_{exp} - G^{lig}_{exp}) \\
=& (G^{holo}_{calc} - G^{holo}_{exp}) - (G^{apo}_{calc} - G^{apo}_{exp}) \\
=& E_{holo} - E_{apo}
\end{aligned}
\tag{1}
$$

where, ΔG_{calc} and ΔG_{exp} are the calculated and experimental binding affinities, $G^{holo/apo}$ denote the free energies of *holo*/*apo* states and E denotes the systematic error with two components, $E_{holo/apo}$, corresponding to differences in the calculated and experimental free energies in *holo*/*apo* states respectively. It is assumed that free energies of solvated ligands (G^{lig}) are identical in calculated and experimental conditions and hence cancel out, leaving no contribution to the systematic errors.

In an ideal scenario, the calculated and experimental free energies should be identical in which both E_{holo} and E_{apo}, and hence E, should be zero. In other words, the systematic error should ideally be zero. However, practically speaking, one or both components of the error are non-zero due to differences in the calculated and experimental free energies in either *apo* or *holo* or both states. This may be caused by several factors leading to discrepancies in calculated and experimental conditions such as conformational differences (including multiple ligand binding modes and differences in tautomeric and/or protonation states), water occupancy in and around the binding site, force field inaccuracies, errors in experi-

ments (including incorrect conversion of observed experimental values into binding affinities, inconsistencies in pH and concentrations) and so on. It should be noted that both error components ($E_{holo/apo}$) are expected to be positive numbers as experimental free energies should correspond to the "global minima" except in case of erroneous experimental values. The overall error (E) may be positive or negative depending on which of its components is dominantly contributing. It is quite common to see ligand-binding-induced conformational changes in the protein structure due to which E_{apo} is the dominant contributor in most cases (when the holo structure is used to initiate calculations for the apo state). This is evident from our dataset where 11 out of 14 systems studied have negative errors (reason why we denote E by ΔG^{prot}_{conf}). However, there may be a few cases where ligand binding induces little or no conformational changes in the protein. In such cases, other factors may dominate leading to positive errors.

Table S2: Summary of ABFE predictions: The number of ligands studied and corresponding values of several statistical parameters - protein conformational free energy (ΔG^{prot}_{conf}), root mean squared error (before and after adjustment) for all predictions as well as Pearson's r (r_p) between ΔG_{TIES} and experimental results - are reported. Unit for energies is kcal/mol.

System	Complexes	EQ				NEQ			
		ΔG^{prot}_{conf}	RMSE	RMSE^{adj}	r_p	ΔG^{prot}_{conf}	RMSE	RMSE^{adj}	r_p
BRD4_1	8	-1.52(0.65)	2.38(0.60)	1.83(0.36)	0.75(0.17)	-2.09(0.55)	2.59(0.52)	1.53(0.28)	0.80(0.16)
BRD4_2	9	-3.41(0.27)	3.51(0.30)	0.81(0.27)	0.80(0.12)	-4.65(0.32)	4.75(0.29)	0.98(0.25)	0.78(0.12)
Bromosporine	22	-2.54(0.33)	2.98(0.34)	1.55(0.26)	0.62(0.16)	-3.14(0.31)	3.47(0.34)	1.46(0.24)	0.66(0.15)
RVX-208	6	-0.19(0.37)	0.92(0.18)	0.90(0.24)	0.34(0.54)	-0.54(0.38)	1.07(0.15)	0.93(0.26)	0.22(0.57)
RVX-OH	6	-0.36(0.16)	0.54(0.09)	0.39(0.12)	0.55(0.11)	-0.53(0.33)	0.95(0.22)	0.79(0.14)	-0.07(0.14)
CDK2	16	3.57(0.24)	3.70(0.20)	0.97(0.17)	0.68(0.12)	1.12(0.29)	1.61(0.20)	1.17(0.16)	0.72(0.11)
CMET	12	-2.62(0.39)	2.95(0.36)	1.37(0.20)	0.78(0.11)	-3.70(0.51)	4.09(0.45)	1.75(0.32)	0.65(0.19)
Galectine	8	-1.57(0.19)	1.66(0.17)	0.54(0.08)	0.70(0.25)	-1.93(0.21)	2.02(0.20)	0.60(0.11)	0.64(0.26)
JNK1	21	1.28(0.22)	1.61(0.18)	0.98(0.11)	0.82(0.05)	0.55(0.25)	1.27(0.22)	1.14(0.17)	0.73(0.09)
P38	34	-2.40(0.15)	2.56(0.16)	0.89(0.12)	0.68(0.10)	-3.64(0.21)	3.84(0.22)	1.25(0.17)	0.63(0.11)
PDE2	21	-1.92(0.28)	2.30(0.22)	1.27(0.16)	0.26(0.20)	-5.58(0.36)	5.82(0.35)	1.66(0.22)	0.34(0.16)
ROS1	24	-7.73(0.33)	7.90(0.34)	1.63(0.20)	0.75(0.09)	-8.70(0.45)	8.98(0.47)	2.23(0.36)	0.57(0.10)
TRKA	16	-7.63(0.40)	7.80(0.39)	1.59(0.25)	0.59(0.27)	-8.60(0.49)	8.82(0.50)	1.93(0.46)	0.48(0.30)
TYK2	16	6.31(0.21)	6.37(0.22)	0.85(0.10)	0.78(0.11)	1.87(0.42)	2.52(0.40)	1.69(0.26)	0.37(0.23)
All	219	-	4.38(0.20)	1.22(0.07)	0.77(0.03)	-	4.94(0.23)	1.52(0.09)	0.70(0.04)

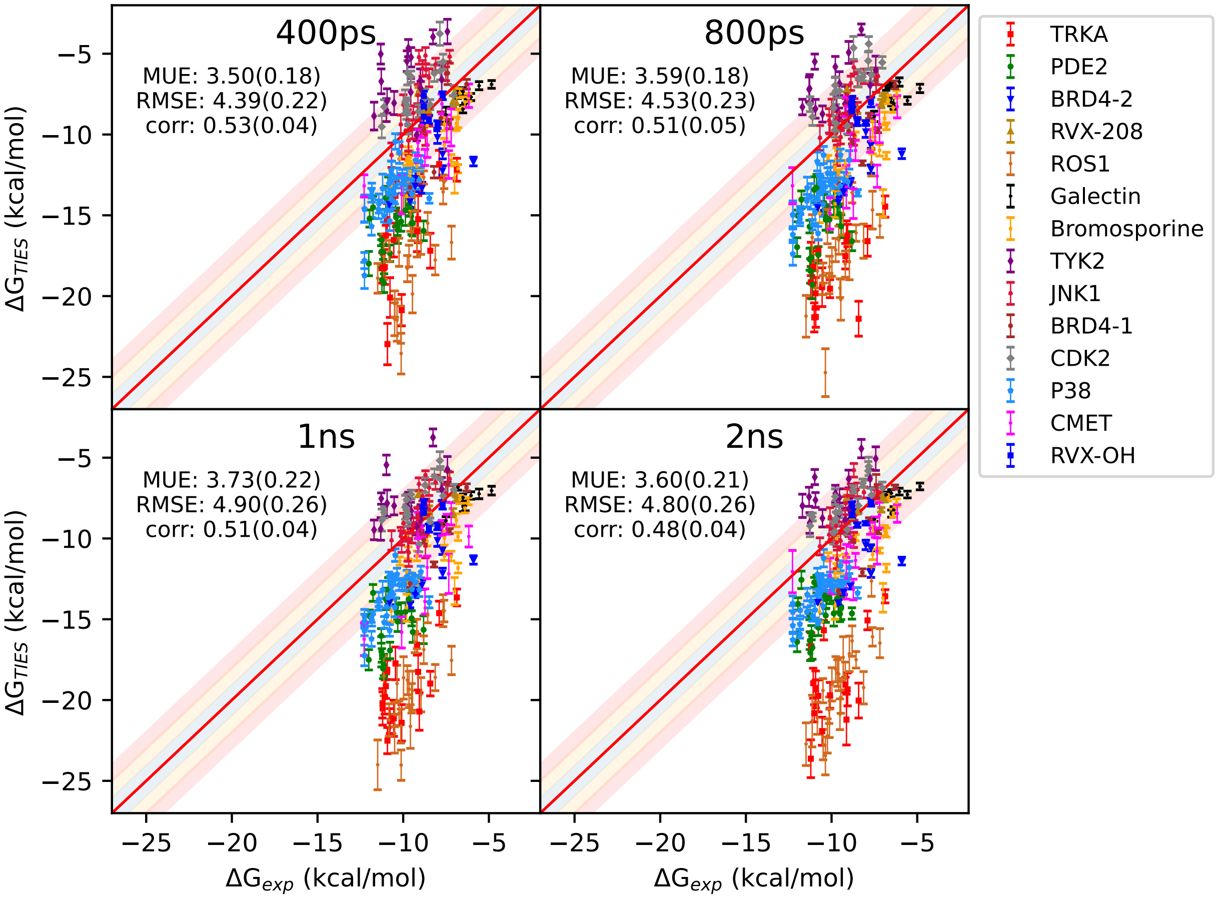

Figure S4: Correlation plots for NEQ predictions using the different transition lengths indicated against experimental data. The solid red line denotes perfect correlation, whereas the blue, orange and red shaded regions represent ±1, ± 1-2 and ± 2-3 kcal/mol ranges.

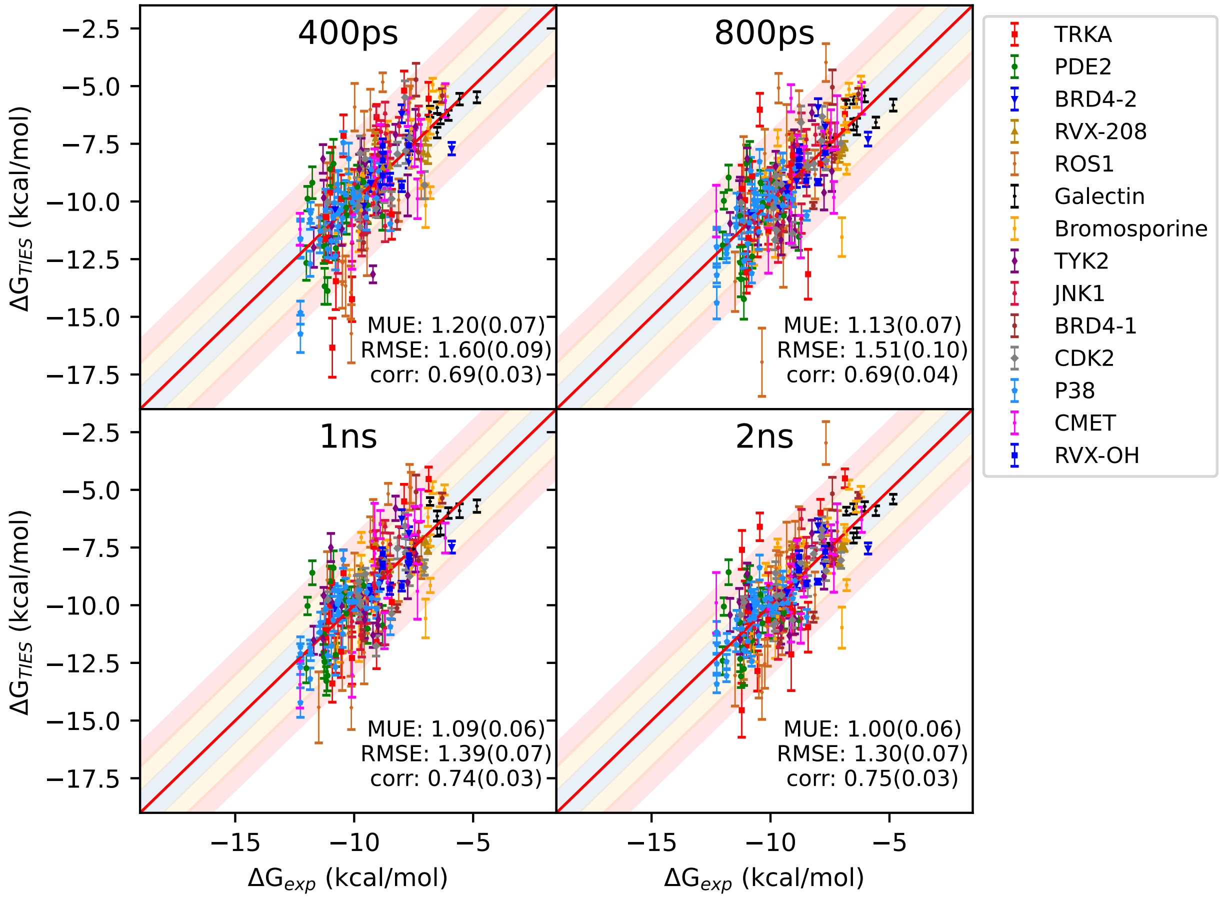

Figure S5: Correlation plots for NEQ predictions after adjusting with ΔG^{prot}_{conf} using the different transition lengths indicated against experimental data. The solid red line denotes perfect correlation, whereas the blue, orange and red shaded regions represent ±1, ± 1-2 and ± 2-3 kcal/mol ranges.

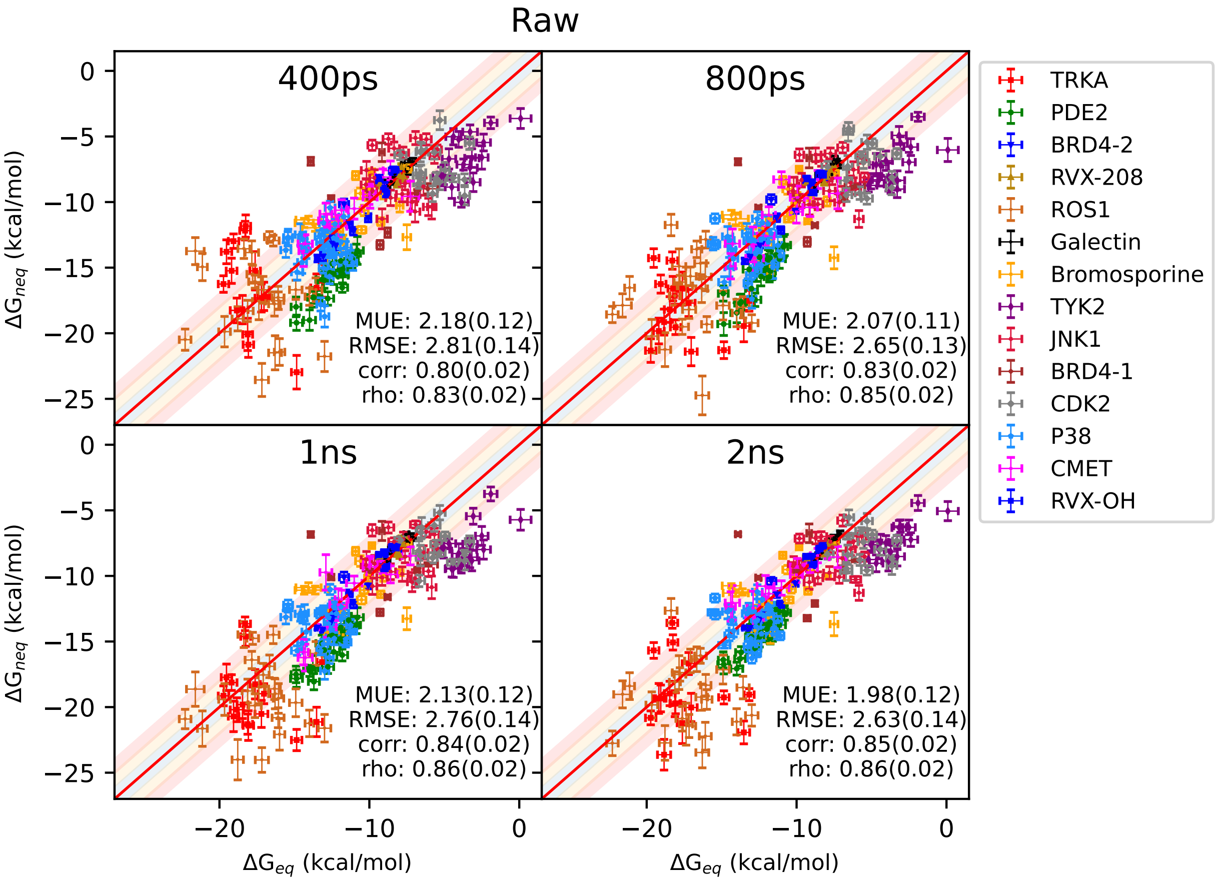

Figure S6: Correlation between NEQ and EQ predictions using the different transition lengths indicated. The solid red line denotes perfect correlation, whereas the blue, orange and red shaded regions represent ±1, ± 1-2 and ± 2-3 kcal/mol ranges.

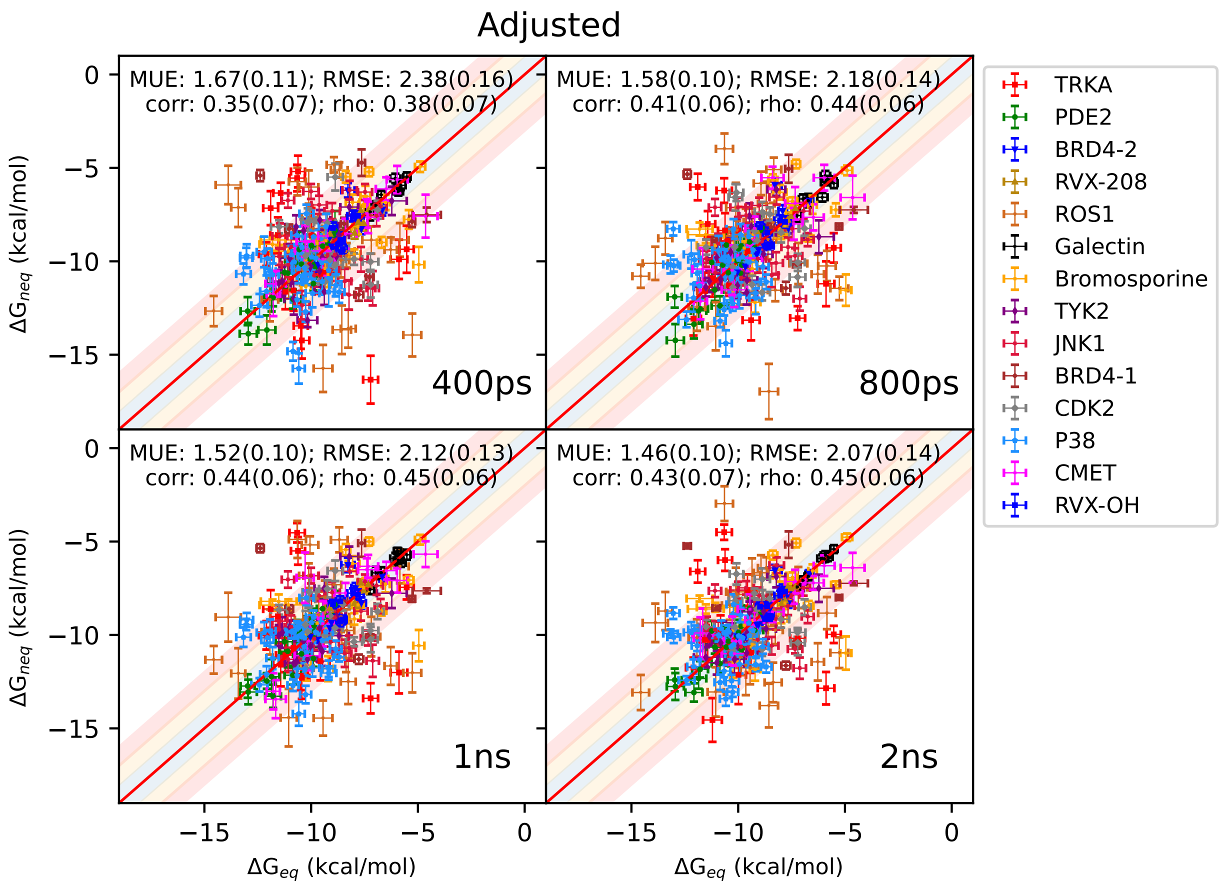

Figure S7: Correlation between NEQ and EQ predictions after adjusting with ΔG_{conf}^{prot} using the different transition lengths indicated. The solid red line denotes perfect correlation, whereas the blue, orange and red shaded regions represent ±1, ± 1-2 and ± 2-3 kcal/mol ranges.

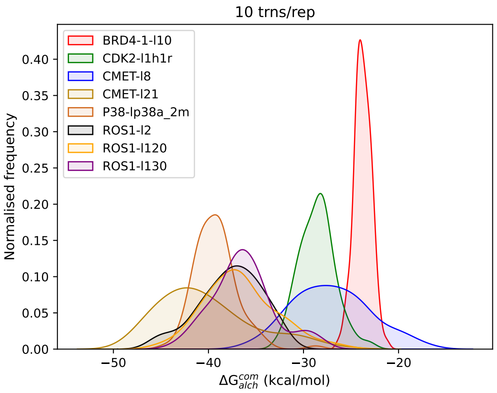

(a) Distributions

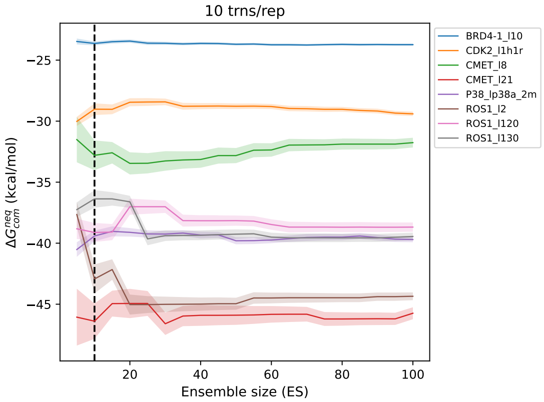

(b) Running averages

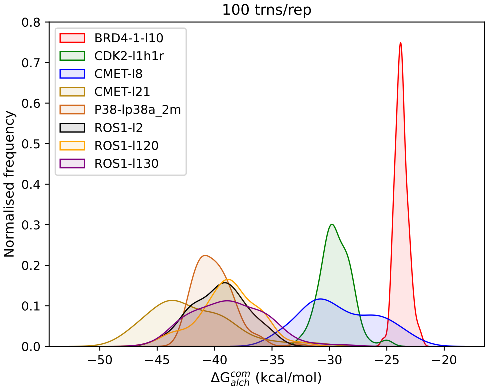

(c) Distributions

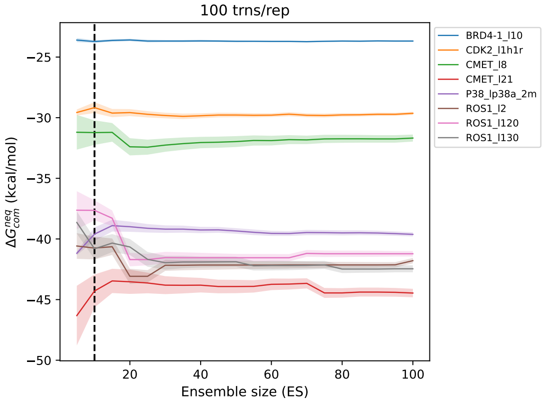

(d) Running averages

Figure S8: **(a, c)** Normalised frequency distributions of predicted ABFEs for the non-equilibrium approach using ensemble size 100 for a subset of ligand-protein complexes depicting their non-Gaussian nature. **(b, d)** Running averages of predicted ABFEs varying with the ensemble size. The dashed line denotes ensemble size 10. The results obtained using 10 and 100 transitions per replica (trns/rep) have been included.

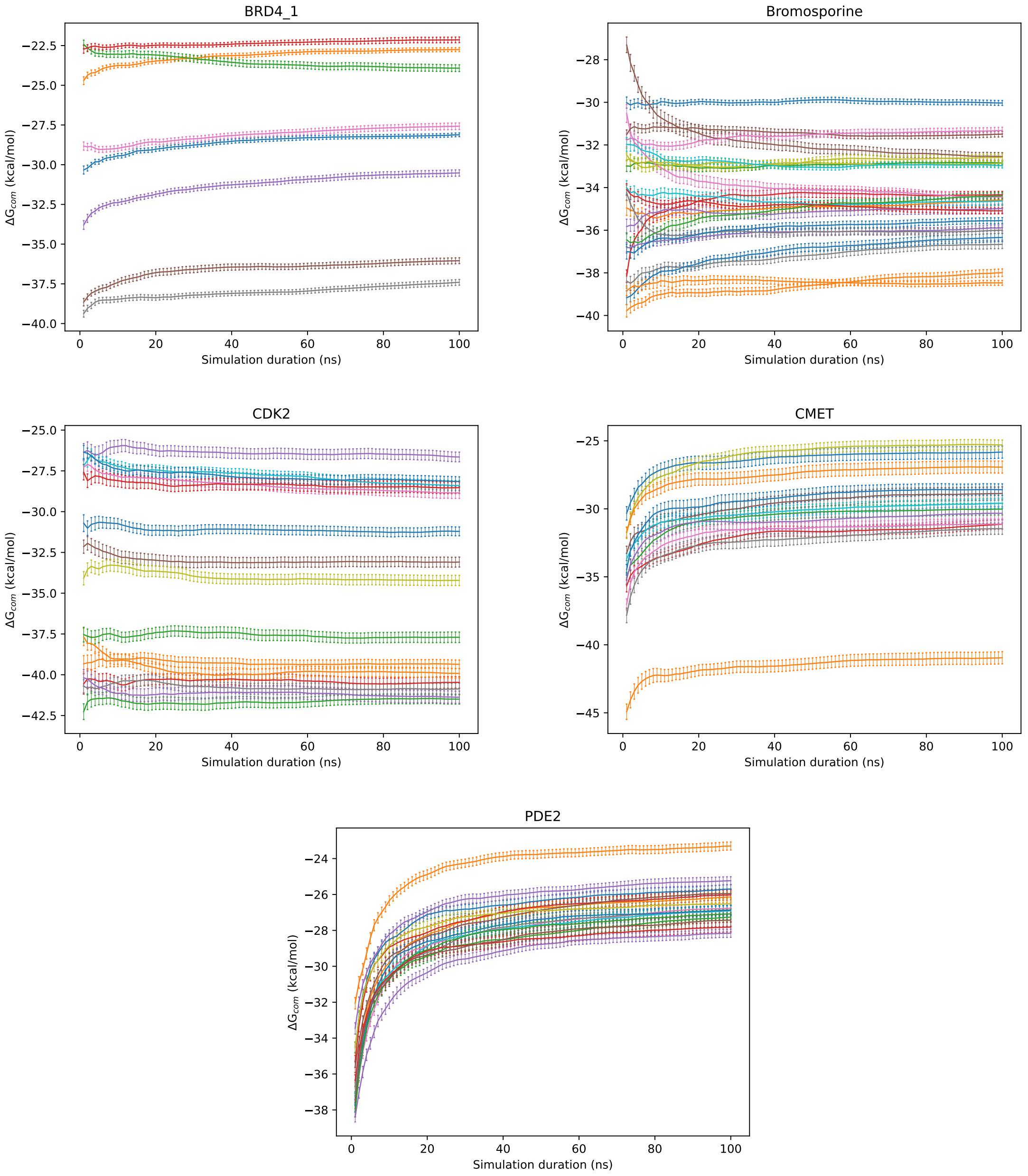

Figure S9: Running averages of ΔG_{com} for 73 different protein-ligand complexes spanning across five different systems for the EQ approach. The replica lengths extend up to 100 ns.

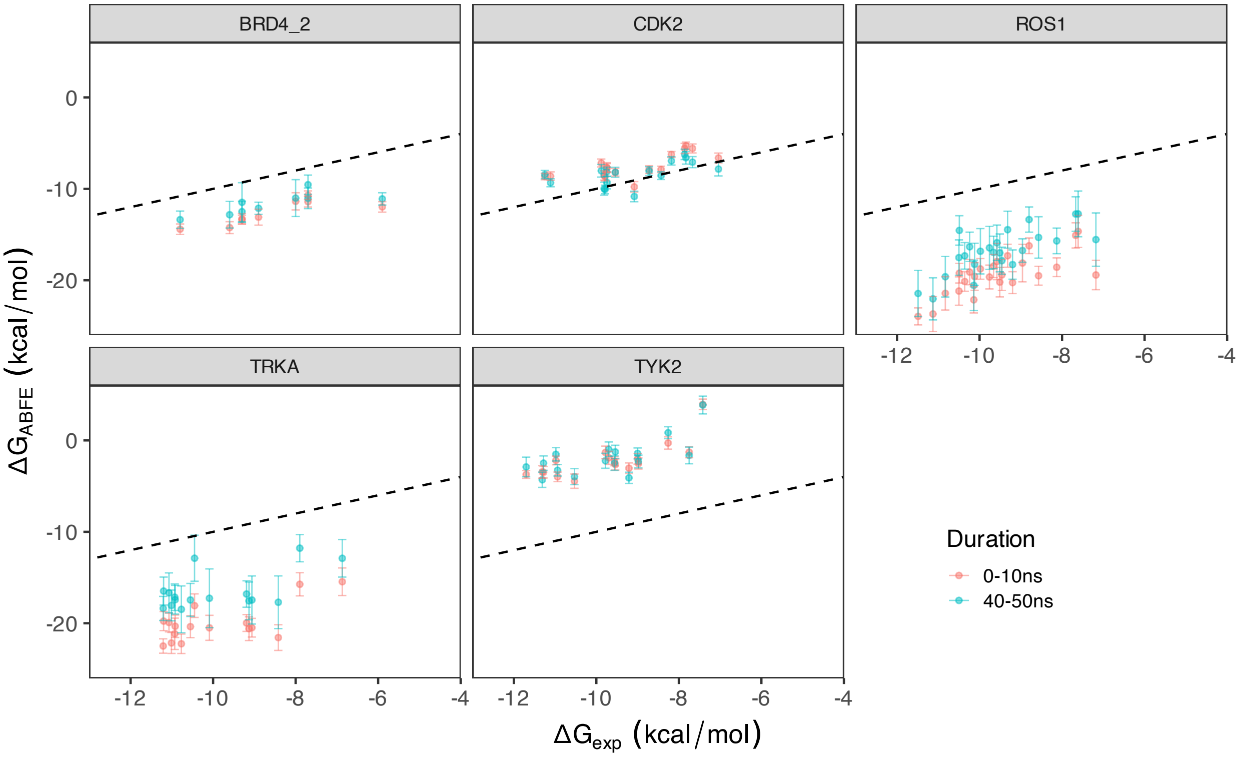

Figure S10: Improvement in ABFE predictions on increasing replica length of the end-state simulations for the NEQ approach. Replica length was increased to 50 ns for 81 complexes (covering five protein systems) for the calculation of ΔG^{com}_{alch}. ABFE predictions from the first and the last 10 ns are compared using 100 transitions and 2 ns transition length in each direction. The dashed line corresponds to perfect agreement between predicted and experimental values.

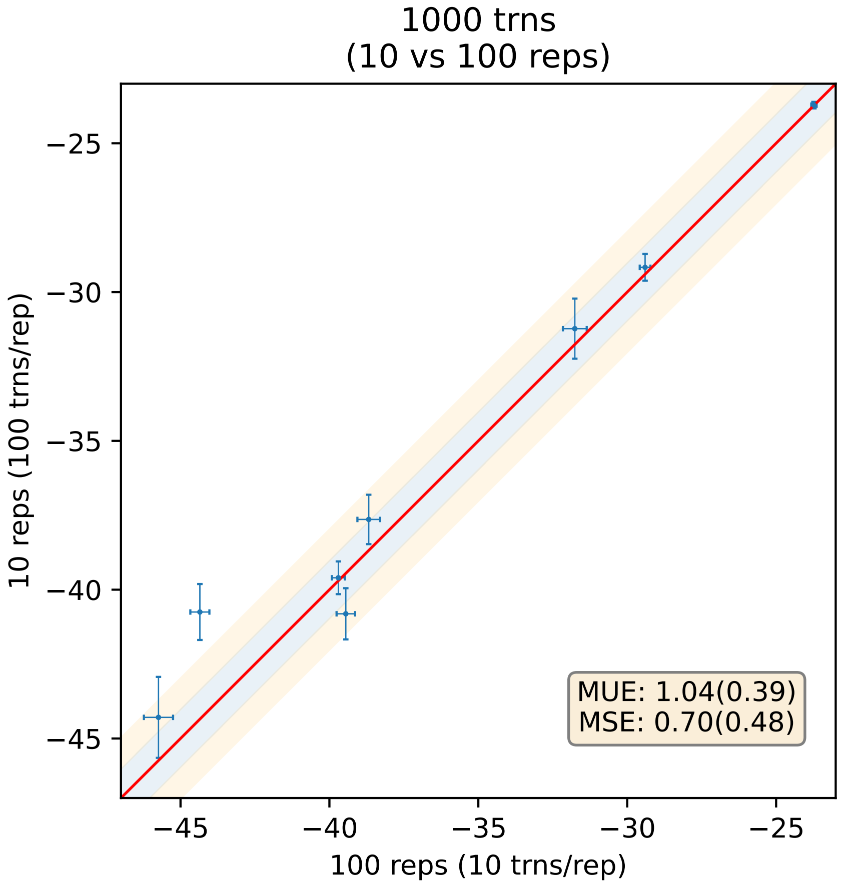

Figure S11: Comparison between ABFE predictions using 1000 NEQ transitions (denoted as “trns”) in each direction with two different combinations: ensemble size 10 using 100 transitions per replica and ensemble size 100 using 10 transitions per replica. The solid red line denotes perfect correlation, whereas the blue and orange shaded regions represent ±1 and ± 1-2 kcal/mol ranges. The mean signed and unsigned errors are included in the inset text box along with associated uncertainties.

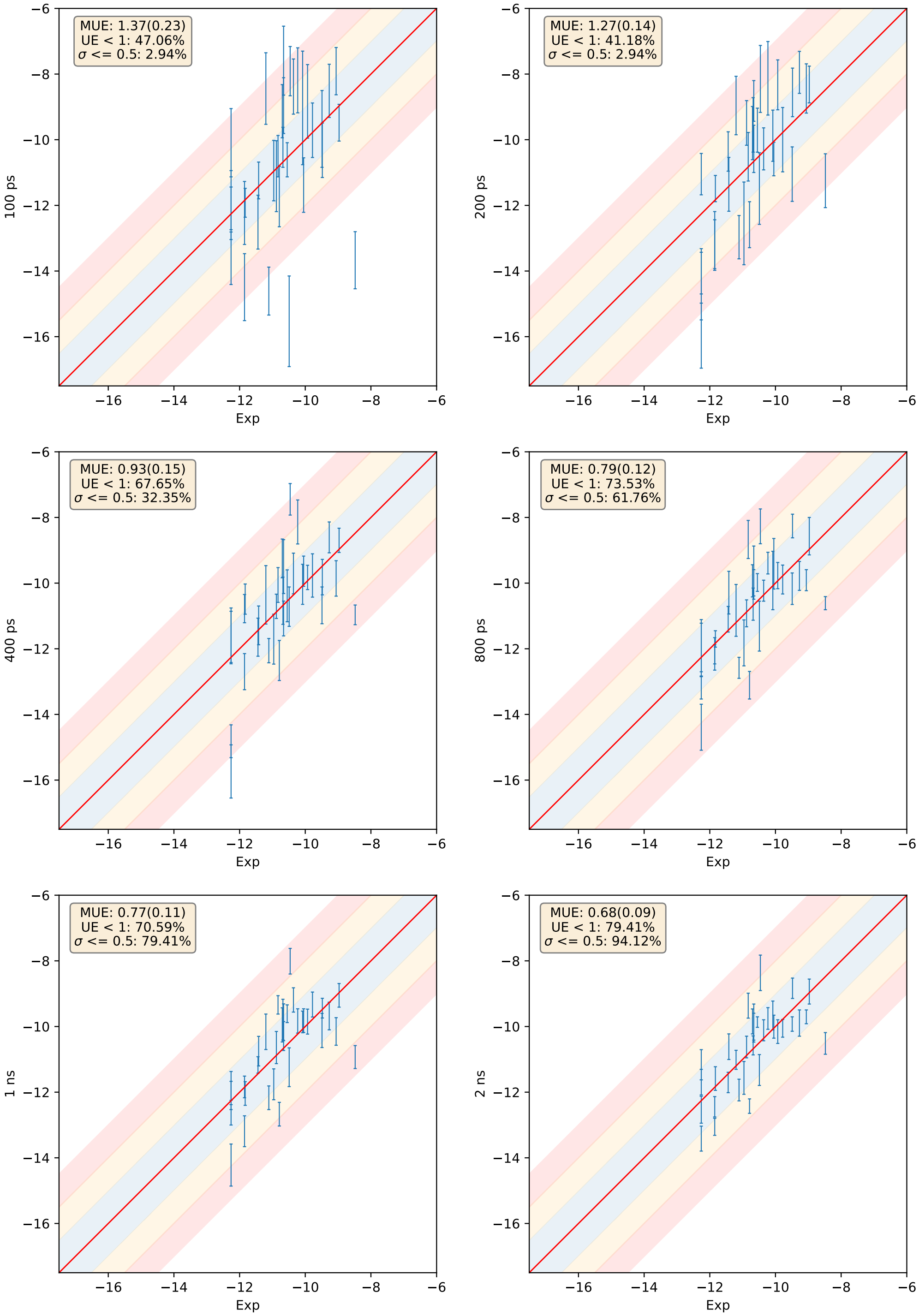

Figure S12: Effect of the length of fast alchemical transitions on the predicted ABFEs. Various lengths are compared against experimental data and corresponding mean unsigned errors (MUE) reported in the textboxes in inset along with associated uncertainties. Fractions of compounds (in % terms) with unsigned errors $<$ 1 kcal/mol and uncertainties $\leq$ 0.5 kcal/mol are also displayed. The solid red line denotes perfect correlation, whereas the blue, orange and red shaded regions represent $\pm$1, $\pm$ 1-2 and $\pm$ 2-3 kcal/mol ranges.

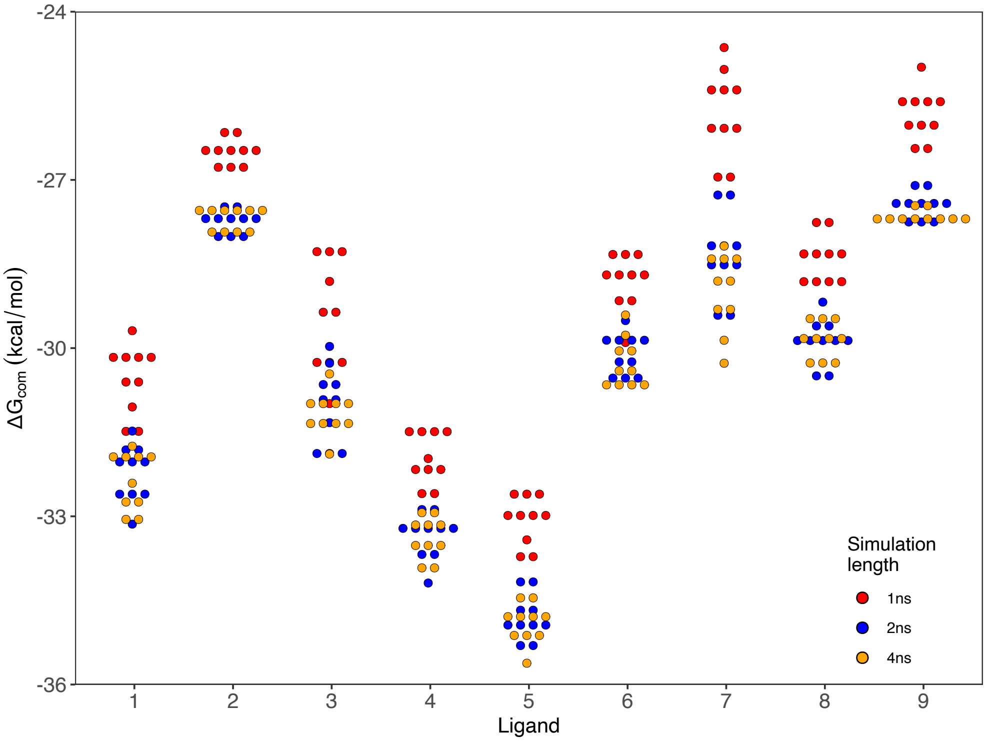

Figure S13: Impact of transition length on and distributions of calculated free energies. 9 compounds from the BRD4-2 system were selected and transition lengths varied (from 1 to 4 ns) for the ligand coupling step in the protein enviroment (ΔG_{com}). Each data point corresponds to a ΔG_{com} obtained using 100 transitions at both end-points starting from evenly selected conformations of a single replica (out of an ensemble of 10) at each end-point. Results using 1 ns transition length are consistently less negative than those using 2 ns and 4 ns transitions, while the latter two yield comparable results. However, the widths of ΔG_{com} distributions remain unaffected by the increase in transition length. Further, most ligands exhibit multimodal character resulting in non-normal distributions irrespective of the transition length used.

Table S3: Effect of the length of fast alchemical transitions on the extent of overlap between the forward and reverse work distributions - an essential requirement for the reliability of free energies predicted using the NEQ approach. Overlap coefficients are defined as the area of intersection of the two work distributions. Four different transition lengths along with our recommended "standard" protocol - 800 ps in the forward direction and 400 ps in the reverse direction (denoted as "600 ps") - are compared. The range of overlap coefficients for each system separately as well as the overall dataset is provided along with the fraction of complexes (in % terms) with zero overlap coefficients in each case. These values are for the ligand transforming in the protein environment. In some cases, the minimum value of the overlap coefficient appears to be zero despite no complexes with zero overlap: this is due to round off error.

System	Complexes	Range					Zero overlaps (%)				
		400 ps	holo	800 ps	1 ns	2 ns	400 ps	holo	800 ps	1 ns	2 ns
BRD4_1	8	0.00-0.03	0.00-0.06	0.01-0.08	0.00-0.08	0.02-0.20	12.5	0	0	0	0
BRD4_2	9	0.00-0.02	0.00-0.03	0.02-0.05	0.02-0.07	0.06-0.14	11.11	0	0	0	0
Bromosporine	22	0.00-0.02	0.00-0.03	0.00-0.04	0.00-0.06	0.01-0.12	36.36	18.18	9.09	4.54	0
RVX-208	6	0.01-0.04	0.02-0.05	0.02-0.07	0.03-0.10	0.05-0.14	0	0	0	0	0
RVX-OH	6	0.01-0.05	0.01-0.07	0.02-0.08	0.04-0.10	0.08-0.20	0	0	0	0	0
CDK2	16	0.00-0.00	0.00-0.00	0.00-0.00	0.00-0.00	0.00-0.01	93.75	93.75	100	75	43.75
CMET	12	0.00-0.00	0.00-0.00	0.00-0.00	0.00-0.00	0.00-0.00	100	100	100	91.67	66.67
Galectine	8	0.00-0.01	0.00-0.02	0.01-0.02	0.01-0.03	0.04-0.06	0	0	0	0	0
JNK1	21	0.00-0.01	0.00-0.02	0.00-0.01	0.00-0.02	0.00-0.04	61.90	38.10	38.10	19.05	0
P38	34	0.00-0.01	0.00-0.01	0.00-0.02	0.00-0.02	0.00-0.06	58.82	38.24	23.53	23.53	0
PDE2	21	0.00-0.05	0.00-0.05	0.00-0.05	0.01-0.05	0.01-0.06	0	0	0	0	0
ROS1	24	0.00-0.00	0.00-0.00	0.00-0.00	0.00-0.00	0.00-0.00	100	100	100	100	100
TRKA	16	0.00-0.00	0.00-0.00	0.00-0.00	0.00-0.00	0.00-0.00	100	100	100	100	100
TYK2	16	0.00-0.00	0.00-0.00	0.00-0.00	0.00-0.00	0.00-0.01	93.75	100	81.25	68.75	31.25
All	219	0.00-0.05	0.00-0.07	0.00-0.08	0.00-0.10	0.00-20	57.08	49.32	45.21	39.73	27.40

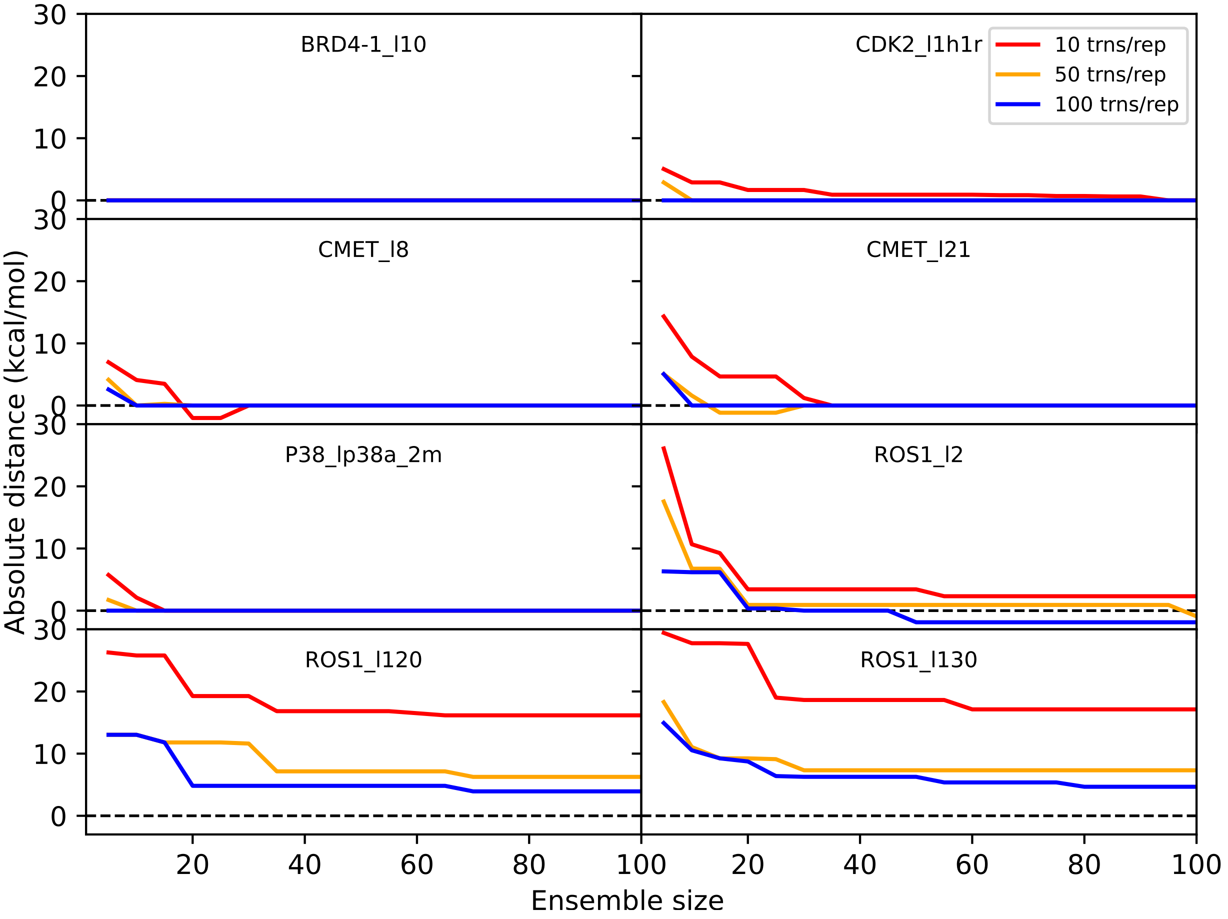

Figure S14: Dependence of distance values (NEQ approach) on ensemble size and number of transitions per replica (trns/rep) for a subset of protein-ligand complexes. The transition length is 2 ns for all cases. The dashed line corresponds to zero distance indicating overlap.

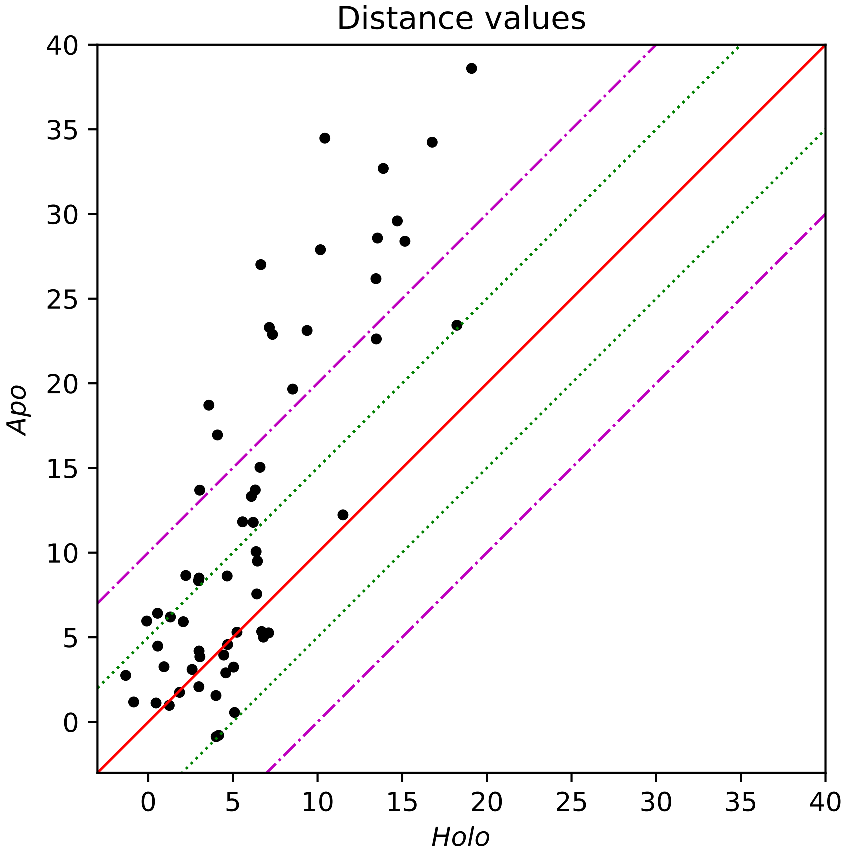

Figure S15: Comparing distance values between *Apo* and *Holo* calculations. All values are in kcal/mol. The solid red line denotes perfect correlation, whereas the broken green and purple lines represent ±5 and ± 5-10 kcal/mol ranges.
